# The effect of cell geometry on polarization in budding yeast

**DOI:** 10.1371/journal.pcbi.1006241

**Published:** 2018-06-11

**Authors:** Michael Trogdon, Brian Drawert, Carlos Gomez, Samhita P. Banavar, Tau-Mu Yi, Otger Campàs, Linda R. Petzold

**Affiliations:** 1 Department of Mechanical Engineering, University of California, Santa Barbara, Santa Barbara, California, United States of America; 2 Department of Computer Science, University of North Carolina, Asheville, Asheville, North Carolina, United States of America; 3 Department of Molecular, Cell and Developmental Biology, University of California, Santa Barbara, Santa Barbara, California, United States of America; 4 California NanoSystems Institute, University of California, Santa Barbara, Santa Barbara, California, United States of America; 5 Department of Physics, University of California, Santa Barbara, Santa Barbara, California, United States of America; 6 Center for Bioengineering, University of California, Santa Barbara, Santa Barbara, California, United States of America; 7 Department of Computer Science, University of California, Santa Barbara, Santa Barbara, California, United States of America; Imperial College London, UNITED KINGDOM

## Abstract

The localization (or polarization) of proteins on the membrane during the mating of budding yeast (*Saccharomyces cerevisiae*) is an important model system for understanding simple pattern formation within cells. While there are many existing mathematical models of polarization, for both budding and mating, there are still many aspects of this process that are not well understood. In this paper we set out to elucidate the effect that the geometry of the cell can have on the dynamics of certain models of polarization. Specifically, we look at several spatial stochastic models of Cdc42 polarization that have been adapted from published models, on a variety of tip-shaped geometries, to replicate the shape change that occurs during the growth of the mating projection. We show here that there is a complex interplay between the dynamics of polarization and the shape of the cell. Our results show that while models of polarization can generate a stable polarization cap, its localization at the tip of mating projections is unstable, with the polarization cap drifting away from the tip of the projection in a geometry dependent manner. We also compare predictions from our computational results to experiments that observe cells with projections of varying lengths, and track the stability of the polarization cap. Lastly, we examine one model of actin polarization and show that it is unlikely, at least for the models studied here, that actin dynamics and vesicle traffic are able to overcome this effect of geometry.

## Author summary

The ability of cells to spontaneously break symmetry and form patterns via networks of protein reactions is a well-studied, yet not fully understood, phenomenon. One particularly useful model system is the mating of yeast cells, where a localization of proteins on the membrane leads to actin cable formation, vesicle traffic, changes in material properties of the cell wall and ultimately to the growth of a mating projection. There is no shortage of mathematical models describing the mechanisms of polarization, yet they are often formulated as differential equations on simple domains. The effect of geometry on the dynamics of polarization has not been systematically explored. There is an interaction between the length scales of polarization and the features of the geometry of the cell that has been under-appreciated and may lead to novel biological insights.

## Introduction

The polarization of proteins during the mating of *Saccharomyces cerevisiae* is a well-studied, yet not fully understood, example of pattern formation in biology. During the mating process, haploid yeast cells respond to a gradient of mating pheromone via a cascade of intracellular protein reactions, culminating in a localization of key proteins on the membrane that facilitate actin cable formation and vesicle transport [1]. Many quantitative models exist, at varying levels of mathematical complexity, for the different levels of polarization in both budding and mating. Broadly, the majority of models have been developed to study the dynamics of the main polarity regulator, the Rho GTPase Cdc42 [2–4], the formation of actin cables and the polarisome [5–7], or the interaction between the two [6, 8, 9]. Reviews of polarization models can be found in [10–13]. The literature for models of polarization in yeast is vast and often contains conflicting results regarding the role of different mechanisms. One key feature that has only recently been addressed is the effect of stochasticity, with some studies having shown that it can be critical for certain models [3, 5]. In particular, stochastic dynamics can more robustly reproduce a highly polarized phenotype and track a moving pheromone input [5].

Stochastic dynamics at the intracellular signaling level has become a standard modeling paradigm in many areas of biology. There are numerous cases where deterministic or mean-field techniques do not capture the relevant dynamics of biological systems [14–16]. Stochasticity is critical particularly when the copy number of a key chemical species is very small, as is often the case within single cells. Many methods exist to numerically simulate stochastic biochemical networks, the most common of which is the Stochastic Simulation Algorithm (SSA) or Gillespie algorithm [17, 18]. One assumption of the SSA is that the system is well-mixed, meaning that the reactants are assumed to be equally likely to be anywhere in the spatial domain. This assumption clearly does not apply in many systems, including our system of interest, polarization in yeast mating. The spatial nature of our system necessitates the use of spatial stochastic methods, of which there are many. One common approach relies on the Reaction-Diffusion Master Equation (RDME) formalism [19–22]. Using RDME techniques, we have studied the protein signaling network involved in polarization in previous works [5, 23, 24]. In particular, we are interested in understanding how these protein signaling networks interact with cell mechanics to yield morphogenetic change, such as the growth of the mating projection.

When the coupling of mechanics and biochemistry in this system is considered, the physical domain of the numerical simulations necessarily becomes time-dependent as the cell changes shape over time. We have previously developed an algorithm to simulate spatial stochastic reactions on time-dependent domains using the RDME formalism [24]. Many existing models [2–5] treat polarization as a Partial Differential Equation (PDE) in simple 1 or 2 dimensional geometries (such as a line or circle). One key feature that is lost in these simulations is the effect that a more realistic geometry can have on these reaction networks. Although recent work has noted a qualitative effect of geometry on simplified models of polarization in yeast mating [25], a thorough characterization of these effects on more complex reaction networks and geometries has yet to be performed. In this paper, we investigate the effect of cell geometry during mating projection growth on recently developed models of polarization in yeast mating. In particular, we simulate 3D spatial stochastic models of polarization in tip-shaped geometries obtained from simulations of the mechanics of the yeast cell wall. We show that this effect is meaningful and needs to be studied further to more deeply understand how biochemistry and mechanics interact to create morphogenetic change, such as the growth of a mating projection.

The effect of geometry on biochemical reaction networks, and in particular on the spatial localization of such models, has been studied in other contexts. Notably, a model of protein localization and cell division in *E. coli* showed that the geometry of the cell can induce pattern formation even in networks with no dynamic instability [26]. The effect of complex geometries on models of polarization for different systems has also been studied and shown to be important [27–33]. For example, cell shape and elongation were shown to be critical for polarization in cell chemotaxis and the speed of response to chemical gradients [27, 28, 33]. Another study revealed that an interaction between cell shape and biochemical regulatory loops with negative regulators can help explain information flow in neurons [32]. Most models of polarization in cells contain both membrane bound and cytoplasmic species, with reactions taking place on the membrane. A critical feature mentioned in the above studies is the surface area to volume ratio of different shapes. This will no doubt have an effect on models of this type, but exactly what the effect will be for a given nonlinear network of reactions is not known.

To address these issues in the context of yeast mating, it is our goal to study the effect of realistic, 3D tip-shaped geometries on current spatial stochastic models of polarization in yeast. To this end, we use computational studies to generate predictions of the effects of geometry on polarization during yeast mating, and compare to experimental results. These simulations have been performed in our software PyURDME [23] which can simulate spatial stochastic dynamics on complex, 3D and time-dependent geometries. Our results certainly do not encompass all current models of polarization or even attempt to quantify all of the possible effects different geometries can have on these models, but rather demonstrate that realistic geometries can have major effects on a general class of polarization models and thus needs to be considered more thoroughly in modeling moving forward. We also raise the question of what mechanisms the cell uses to overcome the apparent destabilizing effect of cell geometry on the location of the polarization cap *in vivo*, and provide some plausible answers.

## Results

### Effect of geometry on models of Cdc42 polarization

**Fig 1:**
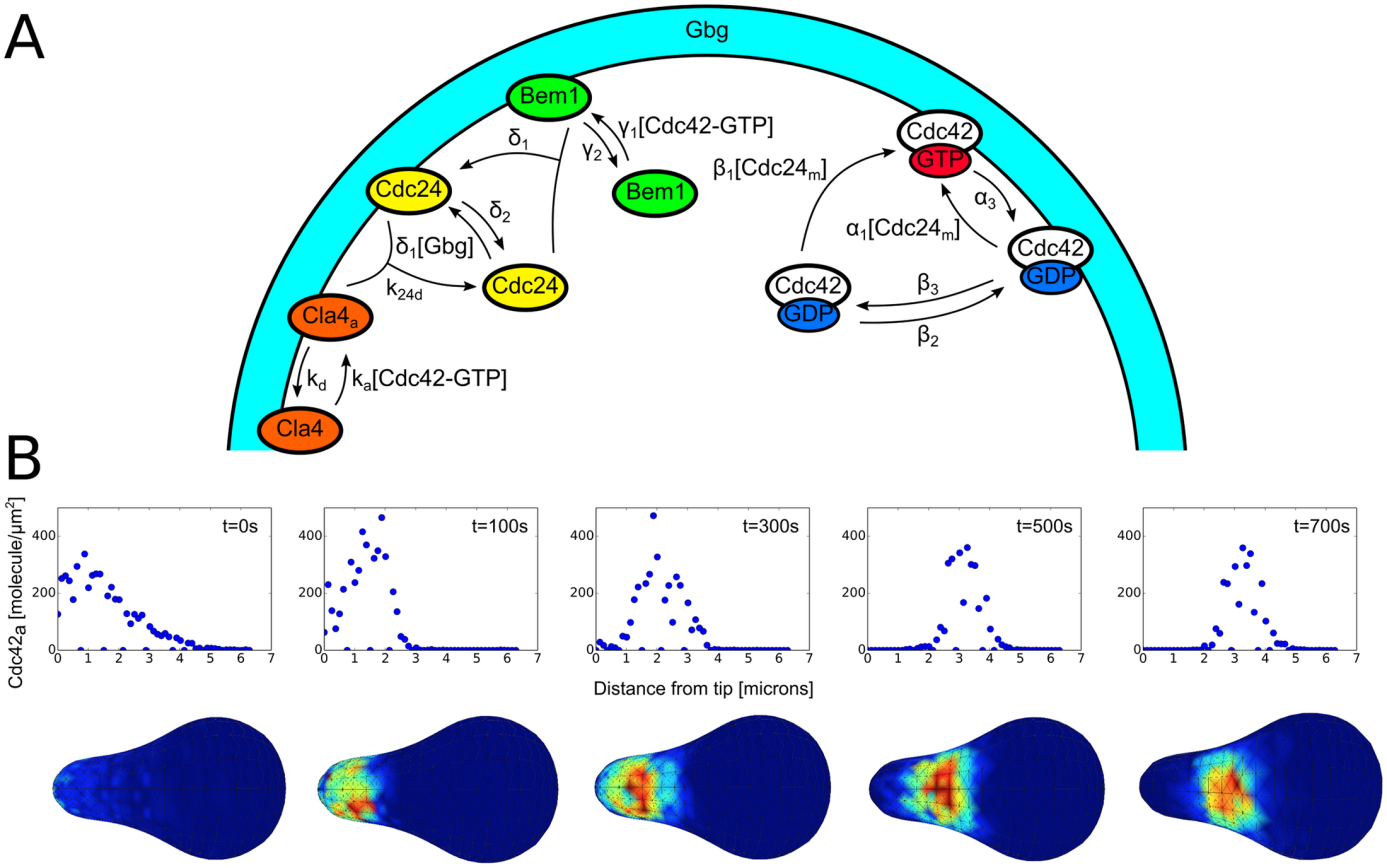
Schematic of Cdc42 model and drifting of Cdc42 cap for one realization. A: Schematic of the detailed, mechanistic model of Cdc42 polarization. Adapted from [4]. Here a uniform input of Gbg is used to model the effect of mating pheromone. B: Visualization of the active Cdc42 polarization cap drifting away from the tip during one realization. The top panel is a plot of the surface density of active Cdc42 versus the distance away from the tip along the perimeter of the shape for various time points (to get a 1D plot, the 3D profile was averaged along the surface of the shape). The bottom panel shows the corresponding 3D visualization of the active Cdc42 distribution. The drifting shown here took place in a matter of 700 seconds.

To study the effect of realistic geometries on the dynamics of models of Cdc42 polarization, we first needed to create computational meshes of such realistic geometries. To do this, we extracted shapes from a model that combines cell wall mechanics and assembly to determine the shape of the cell during projection growth [34]. This model was critical in showing that a feedback between growth and mechanics is needed to stabilize mating projection growth in such systems. Since the timescales of cell wall expansion and growth (cell shape changes) are much longer than the timescales of the molecular reactions involved in cell polarization, we simulate molecular polarization models in a fixed geometry. Our first test was to run a relatively detailed, mechanistic model of Cdc42 polarization adapted from the budding literature [4] (see Fig 1A and the S1 Model in the Supporting Information for details) with polarized initial conditions in a typical tip-shaped geometry. A detailed description of the models and parameters used in this paper can be found in the Supporting Information, along with a more detailed description of the computational methods in the Materials and Methods section. As seen in Fig 1B, the polarization of active Cdc42 can be seen “drifting” out of the tip over the course of 700 seconds. The top panel of Fig 1B shows a time series of a histogram of active Cdc42 density on the surface versus arc-length away from the tip, while the bottom panel shows the same time series with a plot of the full 3D surface concentration of active Cdc42. The polarization cap can be seen to start in the tip of the projection and then drift out (while staying as an intact cap) as time goes on. This model of polarization is known to be stable in time for spherical geometries (see results below and [4]) so this drifting behavior would seem to indicate that there is some clear destabilizing effect on the location of the cap by the geometry itself. This is a crucial result because during mating projection growth, polarization of key proteins must stay in the tip of the projection for proper growth. It is important to note that Fig 1B represents results from only one stochastic realization. To further characterize and understand this result we have run multiple realizations of similar simulations for different geometries, which we discuss further below.

**Fig 2:**
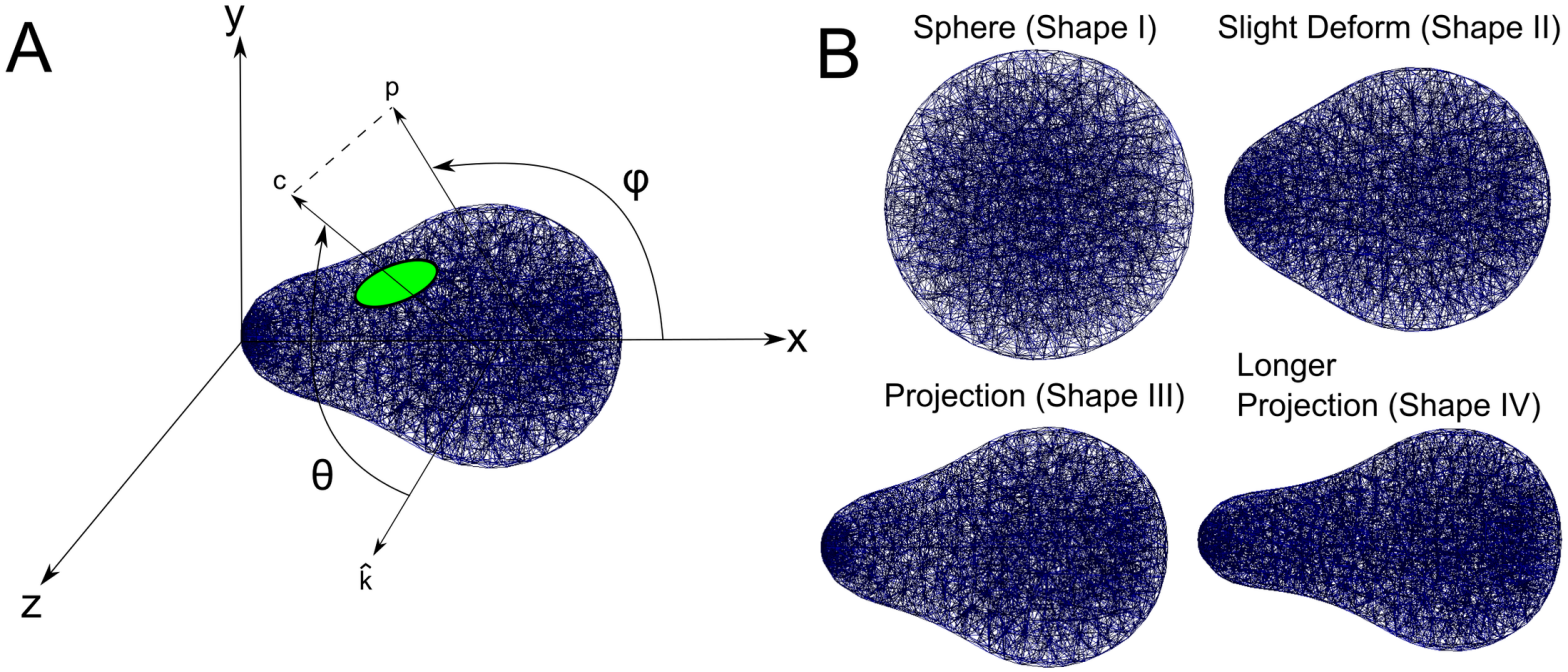
Diagram of angles and geometries used in subsequent simulations. Visualization of the geometries and computational meshes used throughout this study. A: Shown here is an example of the relevant geometry and angle definitions that will be used in later analysis. The green ellipse is meant to symbolize the active Cdc42 polarization cap. The vector *c* is drawn from an origin on the x-axis through the center of the polarization cap and the vector *p* is the projection of *c* onto the xy-plane. The angle *θ* is defined in the usual way in spherical coordinates and is the angle from the z-axis to the vector *c* and is between 0 and 180 degrees. The angle *ϕ* is defined as between the x-axis and *p* and is between 0 and 360 degrees. In this way we can quantify the position of the polarization cap. B: The four computational meshes that will be used throughout this study. They are meant to represent yeast mating projections at different stages of formation. To solve for these shapes, a mechanics solver from [34] was used.

**Fig 3:**
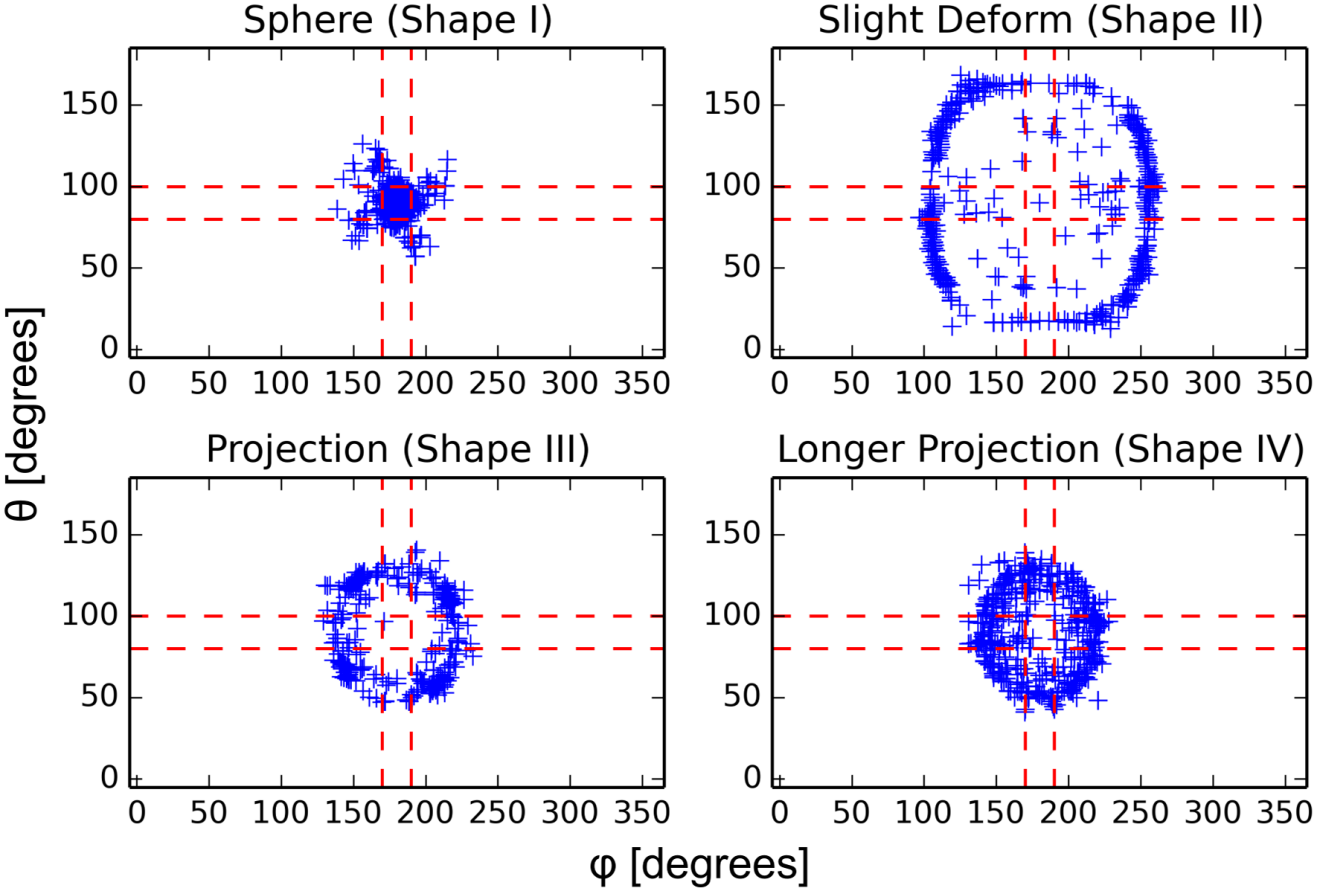
Spherical coordinates of the center of active Cdc42 polarization for multiple realizations with polarized initial conditions. Here the center of the polarization cap is tracked for four different shapes with 800 realizations each. Plotted is the *θ* and *ϕ* coordinates (explained in Fig 2A) of the center of the polarization cap after 1000 seconds of simulation starting from polarized initial conditions at the tip (*θ* = 90° and *ϕ* = 180°). Each point represents the result of one stochastic realization. We can see that for the three irregular tip shapes the polarization cap has drifted away from the tip, whereas for the sphere the cap is stable. The dashed red lines delineate a region of ±10° from the tip, with the red square in the center representing the tip of the projection. The four plots correspond to the four domains shown in Fig 2B. See S8 Fig in the Supporting Information for a plot of *θ* versus distance from the tip.

To more fully characterize the effect of geometry on the dynamics of this model of Cdc42 polarization, we have run similar simulations to that presented in Fig 1B on a variety of shapes. Specifically, we look at four shapes chosen to roughly approximate the different shapes a yeast cell can take during the formation of a mating projection. The computational meshes (again obtained from a simulation of the mechanics of the cell wall [34]) can be seen in Fig 2B. The first set of experiments is equivalent to that of Fig 1B. Namely, we start the simulation with the species polarized in the “tip” of the geometry (for the sphere there is obviously no tip but the polarization is simply started in the same place for each realization) and run multiple realizations for each geometry in Fig 2B. To quantify the end result of each realization, we record the center of the polarization cap (see Fig 2A for visualization) in spherical coordinates after 1000 seconds of simulation. The results of these simulations can be seen in Fig 3 where the spherical coordinates for the center of polarization of active Cdc42 after 1000 seconds are plotted for 800 realizations. The tip of the shape is set to be at *θ* = 90° and *ϕ* = 180° and the dashed red lines in Fig 3 represent ±10° from the tip. For the spherical geometry, the active Cdc42 polarization cap is very stable for the duration of the simulation, while for the other tip-shaped geometries the cap can be seen to drift to a manifold away from the tip (as visualized for one realization in Fig 1B). Moreover, the polarization cap ends on a different manifold for each of the tip-shaped geometries (each realization ends in a ring that is much larger for the “Slight Deform” geometry than for the “Projection” geometries, for example). These results add evidence to the observation that the dynamics of the polarization cap are being significantly impacted by the geometries. Moreover, the polarization cap appears to be stable in a region bounded away from the tip for these projection geometries.

One key feature of the models of polarization that we are using is that they can spontaneously polarize from random initial conditions. To further test the effect of tip-shaped geometries on the dynamics of polarization, we have run a similar set of experiments as those presented in Figs 1B and 3, but start with random initial conditions rather than polarized. Again, the center of the polarization cap was recorded after 1000 seconds and the results of several realizations can be seen in S1 Fig. In the spherical geometry, the polarization cap is distributed randomly over the sphere for several realizations (as expected, as the model has no preference or external bias and polarizes spontaneously) while, again, for the tip-shaped geometries the polarization caps cluster in a manifold away from the tip. Moreover, these polarization sites are the same as those seen in Fig 3, meaning that the polarization cap is stabilizing in the same place for both polarized and random initial conditions. This result adds evidence to the claim that these locations in the tip-shaped geometries are globally stable for this particular model of Cdc42 polarization. Again, this is critical in that it implies that simply changing the geometry of the domain can drastically affect the dynamics of polarization. There is clearly an interplay between the geometry, diffusion in the bulk and on the membrane, and the reactions that is leading to this effect on the location of the polarization cap, which we will discuss more later.

**Fig 4:**
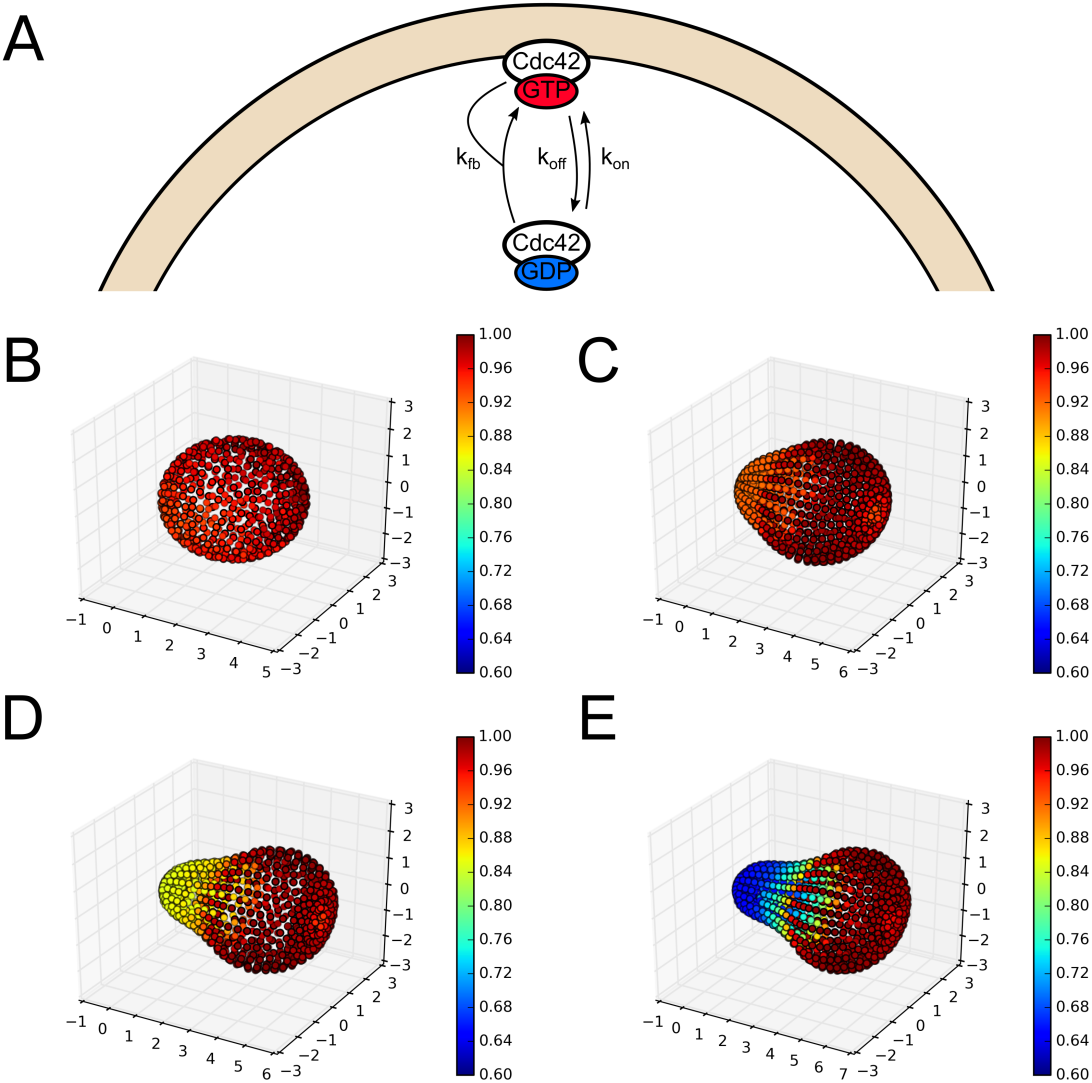
Effect of geometry on a simplified model of Cdc42 polarization. A: Schematic of the simplified model of Cdc42 polarization. Adapted from [3, 35]. B-E: The normalized time average of the concentration of membrane bound species for each voxel is shown for four different geometries. The model presented in [3] and the S2 Model was run for 100,000 seconds for each geometry in Fig 2B with a total of 200 realizations for each. As mentioned above, this model exhibits stochastic clustering that is dynamic in time, with clusters forming and breaking up. A clear bias can be seen, with stochastic clusters systematically staying out of the tip of the projection, which is qualitatively similar to the results presented in Fig 3.

In addition to the more detailed mechanistic models of Cdc42 polarization studied above, there are many simplified models of Cdc42 polarization that are simpler mathematically and preserve some of the key features of polarization. We have also looked at the effect of tip geometries on these simplified models of polarization. In particular, we studied the effect of geometry on the simplified model presented in [3, 35]. This model contains only the membrane bound and cytoplasmic versions of Cdc42 with three reactions: attachment and detachment from the membrane and recruitment of cytoplasmic species by membrane bound species (see Fig 4A and the S2 Model in the Supporting Information for details). Interestingly, this model has been shown to exhibit clustering behavior only when modeled stochastically, as opposed to deterministically. Again, the advantage to this model is that it is much simpler in the number of species and reactions, while the disadvantage is that it does not fully capture the polarization dynamics seen in cells. Specifically, this model will lead to sporadic clusters that are not stable in time but rather form and break up dynamically and indefinitely. Thus, a different metric is needed to quantify the effect of geometry than was used for previous models. For this reason, we have run the S2 Model in the same four geometries presented in Fig 2B for 100,000 seconds and simply averaged the amount of membrane bound Cdc42 in each voxel over time for several realizations. The results can be seen in Fig 4 below. Again, there is a clear qualitative effect of these geometries on the dynamics of this simplified model. Namely, the clusters are seen in Fig 4C–4E to systematically stay out of the projection. The overall effect of some negative interaction between the tip geometry and polarization is preserved. Both of these models show that there is a clear effect of geometry on the dynamics of polarization, and more specifically that there is a tendency for polarization to avoid the projection in these geometries. This further raises the question of how cells overcome this effect *in vivo*, which we will explore below.

### Effect of large deformations and changes in density on models of Cdc42 polarization

**Fig 5:**
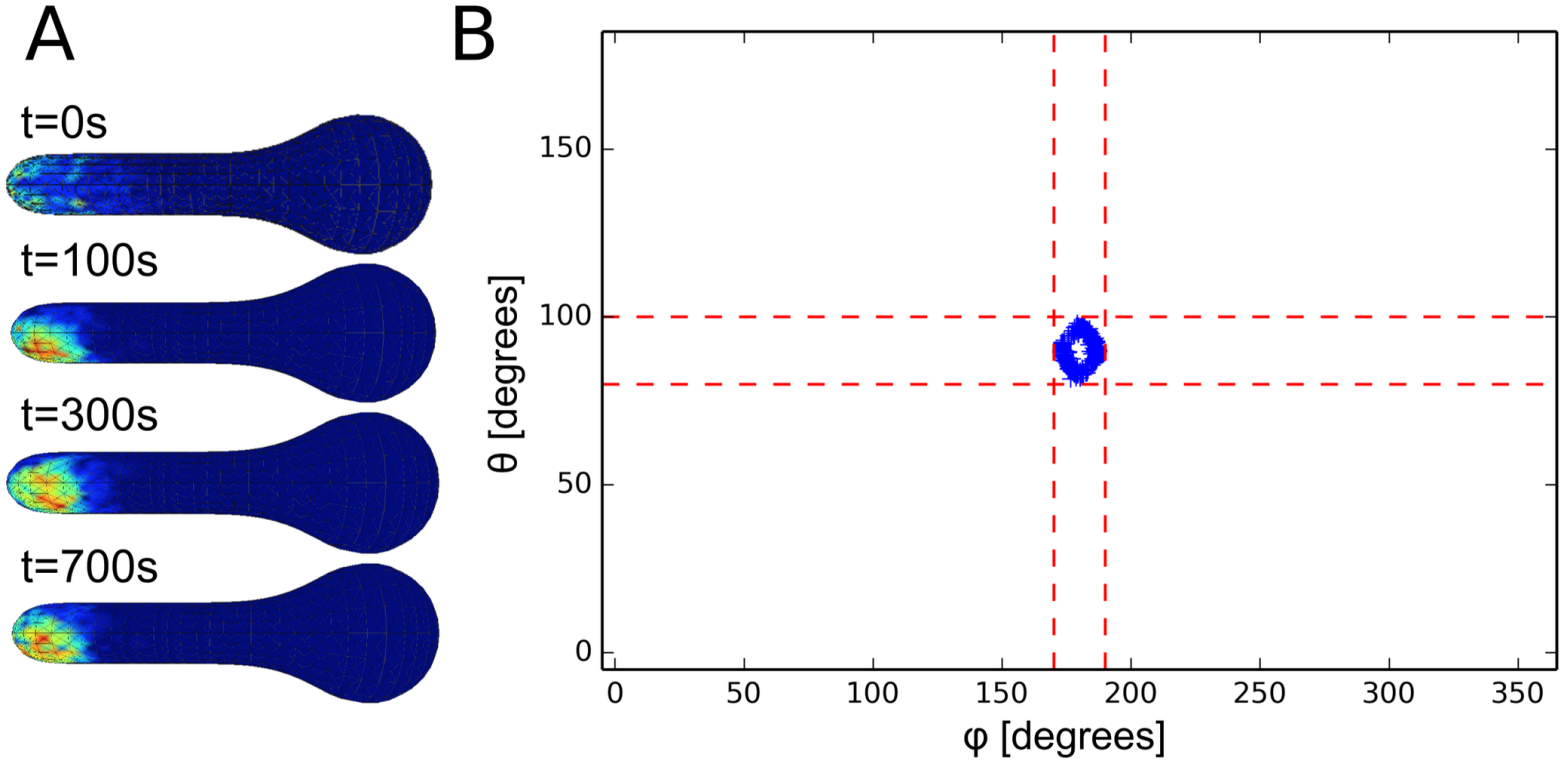
Effect of long projection on Cdc42 polarization. Visualization of effect of a long projection geometry on Cdc42 polarization. A: Time series for one realization of Cdc42 polarization starting from polarized initial conditions. The polarization cap can be seen to drift slightly away from the tip but still stay in the projection. B: Summary of where the polarization cap ends up starting with polarized initial conditions for 400 realizations after 1000 seconds, similar to previous results. The cap can be seen here to be much more stable in the projection compared to the shorter projection geometry results presented above.

In addition to the geometries studied in the previous section, we investigated the effect of geometry on the same reaction-diffusion models, for more pathological geometries. Specifically, we simulated the Cdc42 polarization model on geometries with projections much longer than those seen in WT cells. We will discuss the physical relevance of these shapes when comparing to experimental results in the next section. We first ran a computational experiment similar to that presented in Fig 3, but on a long projection geometry. The results of this simulation can be seen below in Fig 5. Surprisingly, when starting with the model polarized in the tip of the projection, the polarization cap was in fact more stable in the tip of the projection. This is in contrast to the results presented above with much shorter projections. Presumably, this is due to the fact that there are multiple meta-stable points for the polarization cap throughout the geometry. In the case of the short projection, the size of the polarization cap (set by the specific reactions and diffusion rates of the model) is on the same order as the spatial distance between the tip and the more stable position (the neck of the projection in this particular case). While for the longer projection, the size of the polarization cap is much smaller than the length of the projection. We will discuss these results further below.

**Fig 6:**
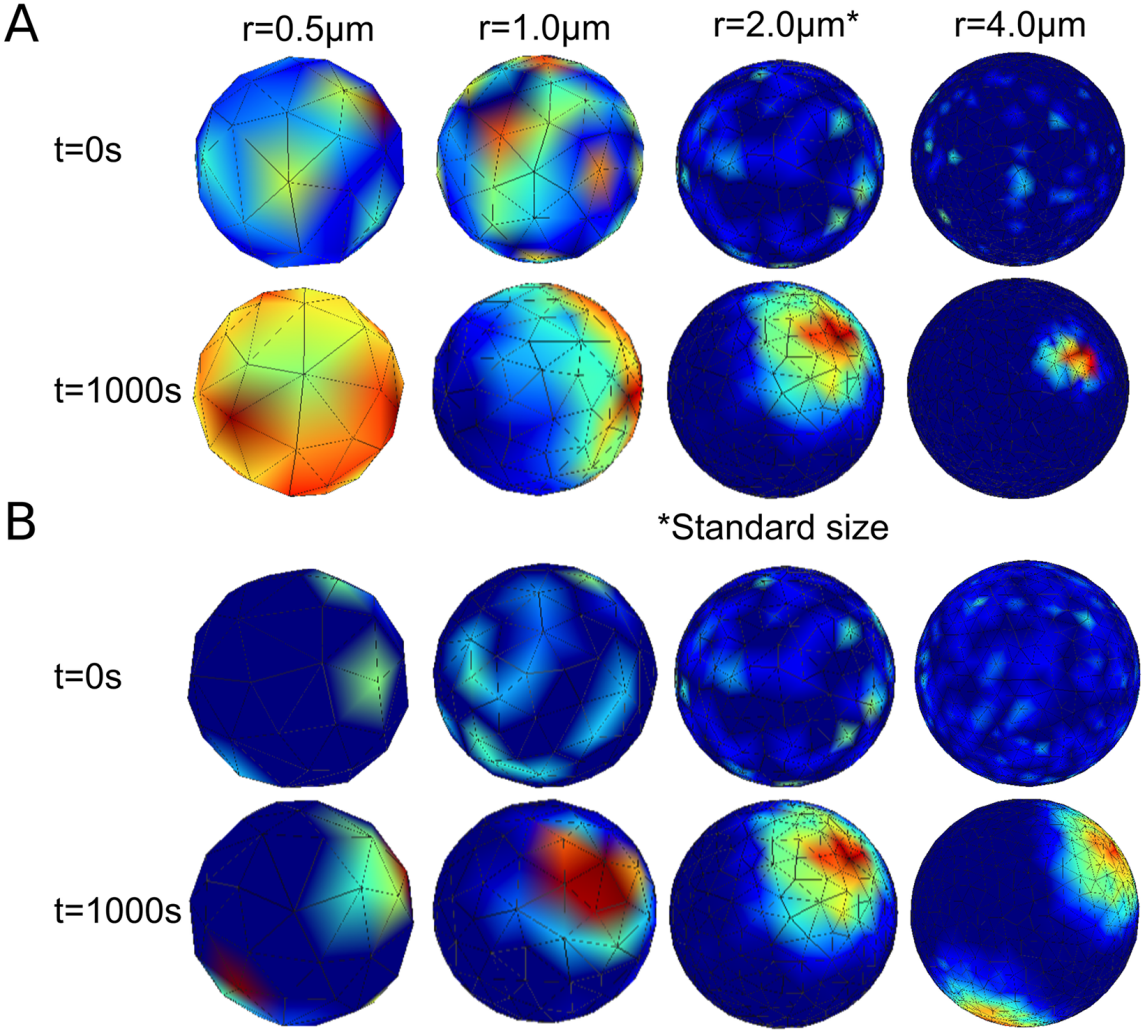
Effect of constant density versus constant molecule count for various spheres. Visualization of effect (for one stochastic realization) of having either a constant density or a constant number of molecules for spheres of varying radii. A: Simulations on spheres with radii *r* = 0.5, 1.0, 2.0, 4.0*μm* are run for 1000 seconds (starting with randomly scattered initial conditions) each with a constant number of molecules, set to be the same number of molecules as the base simulation (for a sphere of *r* = 2.0*μm* with molecule counts given in the S1 Model in the Supporting Information). For each sphere there is consistently one polarization cap with a certain associated size. For the small sphere the polarization cap is bigger than the sphere itself, thus it appears uniform. For the other spheres there is one polarization cap. B: Here the density is held constant (equal to that of the base simulation) rather than the molecule count. Interestingly, different dynamics can be seen. For example, the large sphere has two fully formed polarization sites as opposed to one. Presumably, the increase in the size of the domain and the number of molecules leads to the possibility of two (or more) polarization sites that will possibly compete and merge given enough time.

A related, yet quite distinct, issue to changes in geometry is changes in volume of the cell and density of the species. It is well known that these models, in general, can have very different dynamics with varying density of some or all of the species. In fact, the model presented originally in [3] and above in Fig 4A was shown specifically to be a density dependent switch. That is, stochastic clustering fails to occur below some critical density due to all of the molecules being in the cytoplasm at steady state, yet there exists a range of critical densities that exhibit clustering. Eventually, if the density is increased enough, clustering is lost because so many molecules will be on the membrane at steady state that it effectively creates a uniform distribution. The key challenge here is to isolate the effect of geometry from the effects of changes in density. To investigate the role of density in the polarization of Cdc42 for the S1 Model, we ran a series of simulations on spheres of different radii keeping either the number of molecules, or the density, constant. The results of these simulations can be seen below in Fig 6. For the constant molecule simulations there seems to be a consistent result. Namely, there appears to be one (and only one) polarization cap with an associated length scale or size. For the small sphere, the size of the polarization cap is larger than the sphere itself thus it appears uniform. For the constant density simulations, there is a variety of behaviors. Notably, the larger sphere has two distinct polarization sites. This is surprising in that the original model in [4], from which the S1 Model is adapted, explicitly noted that, at the base values, it is important that only one broad polarization site formed and then narrowed, as opposed to some other models that show competition between clusters (for example in [2]). This shows that in addition to cell geometry, whether the cell fixes density or number of molecules also affects polarization. As for the results presented above in Figs 3 and 4, these geometries deviated only 10 − 15% in volume from the sphere, as opposed to the large scale changes seen in Fig 6 and were run with a constant number of molecules as opposed to a constant density. For completeness, these simulations were also run with a constant density and the overall results were similar (see S4 Fig for details). As mentioned above, the density of molecules throughout the spatial domain can have an important effect on these models. To look at the effects of changes in density independently from changes in overall shape we have run a series of simulations on spheres of different radii. The results in Fig 3 and S4 Fig, which deal specifically with the effect of changes in overall shape, and the results in Fig 6, which deal with the effects of changes in density without changes in overall shape, combine to try and disentangle these two related issues. We will discuss these issues further below. These results also raise some interesting questions about how the cell regulates polarization processes to achieve certain functions. For example, a previous work has looked into the possibility of the cell using changes in density to sense cell size or trigger cell cycle transitions [36]. It might be reasonable to ask if the cell uses geometry effects to its advantage in any way.

Our results show that there is an important and noticeable interaction between cell geometry and the models we have considered here. This is important because typically, more realistic geometries have not been considered when models of polarization have been proposed. One interesting prediction here is the difference in stability of an already formed polarization cap for different geometries. Namely, the polarization cap is stable in the sphere, unstable in a short projection and stable in a long projection (long as compared to the size of the polarization cap itself). This is a result that we attempted to observe experimentally. Prompted by these simulation results, we wanted to see to what extent this phenomenon could be observed *in vivo*. We describe our experimental set up and comparison to our results in the next section.

### Comparing to experiments

**Fig 7:**
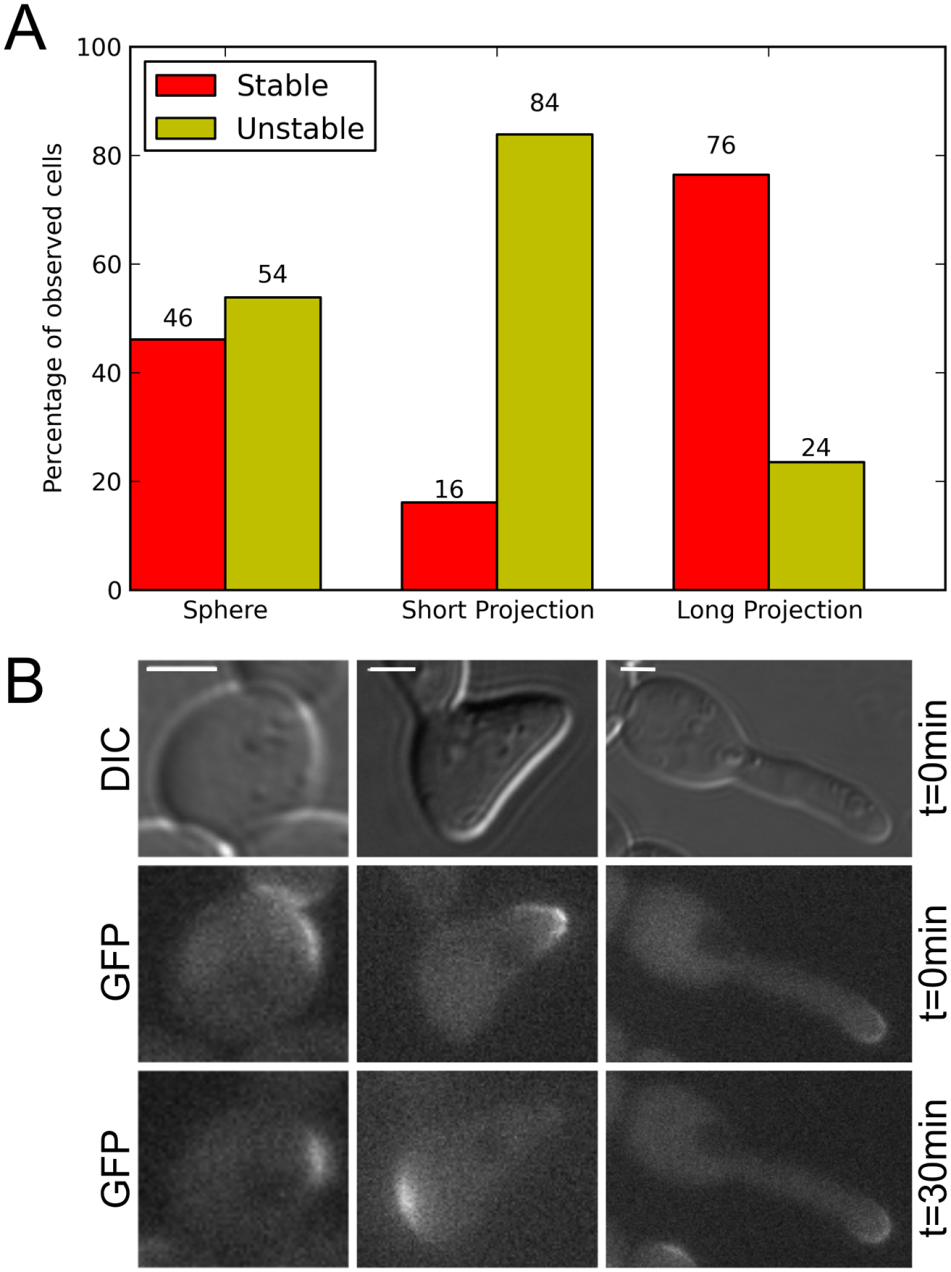
Experimental observations of stability of polarization for different geometries. A: *cla4*Δ cells were exposed to *α*-factor for varying amounts of time to observe a series of shapes of projections: spherical (n = 26 cells), short projection (n = 31 cells) and long projection (n = 34 cells). Cells were then characterized as either stable or unstable by comparing the spatial location of the polarization cap immediately after the application of LAT-A and 30 minutes after. If the polarization cap was in the same location after 30 minutes with no actin cables (due to the LAT-A) then the cell was characterized as stable, otherwise it was unstable. The trend of spherical cells and long projection cells being more stable than shorter projection cells is in line with our theoretical prediction. The differences in the percentage of stable polarizations in the short projection cells versus the spherical (p < 0.05) and long projection (p < 0.001) cells were statistically significant by the chi-square test. B: DIC and Fluorescent images of GFP tagged Ste20 right after the application of LAT-A (t = 0min) and 30 min after for a representative cell of each shape. The spherical and long projection cells here show that the polarization cap stays in the same spatial location throughout the experiment while the short projection cell re-polarizes in a different location. We predict that this instability of the shorter projection cells is due to an interaction between the geometry and the dynamics of the Cdc42 polarization network. All scale bars here represent 2 *μm*. Time-lapse videos can be seen in S1–S3 Movies in the Supporting Information.

While the main thrust of this work is to elucidate the effect of different geometries on various mathematical models of polarization, we wanted to test some of the overall predictions experimentally. To this end, we devised an experiment that attempts to replicate the conditions in the modeling results presented in Fig 3. The main prediction we are testing is that, in the absence of vesicle transport, the Cdc42 polarization cap will be stable in a sphere, unstable in a short projection and stable in a long projection. To achieve long projections, we constructed a *cla4*Δ strain. Cla4 is present in some models of Cdc42 polarization and is thought to negatively regulate Cdc24 via phosphorylation [37, 38]. As a result, some *cla4*Δ cells possess elongated projections, whereas others adopt a more typical morphology. To observe the three different shapes of interest, we allowed *cla4*Δ cells marked with Ste20-GFP (a reporter for active Cdc42) to grow in *α*-factor for varying amounts of time and form different length projections (Materials and methods). Once the mating projection was formed, the cells were treated with latrunculin A (LAT-A) to depolymerize actin cables. This, presumably, leaves the cells with only the Cdc42 polarization pathway for polarization in a tip-shaped geometry, analogous to the models. The GFP-tagged polarization site was then tracked over 30 minutes. We observed cells of each shape: spherical, short projection and long projection. For each group, we categorized the polarization site as either stable (i.e. it stayed in the same spatial location after the addition of LAT-A) or unstable (i.e. it polarized in a different location after the addition of LAT-A). The results of this experiment can be seen in Fig 7.

As predicted, the polarization site was more stable in spherical and long projection geometries when compared to shorter projections. While overall this is a qualitative result, it does suggest an interesting interaction between the dynamics of Cdc42 polarization and the shape of the cell in both simulations and experiment. It is difficult to completely isolate the effect of geometry on polarization, as many processes are disrupted in the absence of actin cables, but we think that looking at three different shapes of cells under similar circumstances provides evidence for an effect of geometry on the dynamics of polarization. It should be noted that the experimental results for the spherical cells are not as stable as the simulation and there are multiple possibilities for why this is the case. It could be simply due to the fact that even cells that were categorized as spherical in the experiment were not perfectly spherical as in the model, but in fact ellipsoid which, based on our previous results, could have some effect on the stability of polarization. Despite these potential issues, the experimental results observed for the three classes of cells are consistent with the model simulations. Overall, there is a complex interaction between the nonlinear reaction-diffusion process and the geometry. It is plausible that there is some bias away from the tip for shorter projections due to the size of the polarization cap being on the same order as the projection length. This is an interesting result because it raises the question of how the cell overcomes this effect *in vivo*. We explore some possibilities in the next section.

### Possible mechanisms to overcome the effect of cell geometry

The results above raise one seemingly important biological question for the interaction of geometry and a variety of reaction-diffusion models of Cdc42 polarization: how does the cell overcome the bias of the Cdc42 cap away from the tip during projection growth? This effect was observed simply by simulating various models of Cdc42 polarization in realistic geometries. A clear first hypothesis is that it is somehow the actin network and vesicle transport that is overcoming this effect. This is relatively difficult to test in a meaningful way simply because the methodology for modeling actin cable formation and vesicle transport in the literature is so diverse. The conclusions about the interaction of the two parallel pathways of reaction-diffusion Cdc42 polarization and actin-mediated vesicle transport are disputed. For example, one major question is whether vesicle delivery provides positive [8] or negative [6] regulation of polarization. The main issue pertains to the role of each pathway in the establishment and maintenance of the polarization site. It is likely that the diversity of opinion arises because of the vastly different ways in which vesicle transport is modeled mathematically. Nonetheless, to try to test this hypothesis, at least initially, we have coupled the previously discussed model of Cdc42 polarization to a model of actin dynamics presented in [5] (see the S3 Model in the Supporting Information for details). The key to this model of polarization is that it is mechanistic and relies on stochasticity to faithfully replicate biological data from mating yeast cells. This combination of a fully dynamic model of Cdc42 polarization and a model of the polarisome is novel.

To investigate the hypothesis that actin-mediated vesicle transport could possibly overcome the negative effect of tip-shaped geometries, we have run similar simulations to those presented in Fig 3. Namely, we ran the fully coupled Cdc42 and polarisome model with polarized initial conditions and tracked the polarization cap of both active Cdc42 and Spa2 in the four shapes shown in Fig 2B. The results can be seen in S2 and S3 Figs. Again, as with the Cdc42 polarization above, the polarization cap tends to end away from the tip in these geometries, while it is relatively stable in the sphere. To test this conclusion in an even stronger setting, we fixed the active Cdc42 profile as polarized in each geometry and simulated the polarisome model with this fixed input. Even in this case, with the Cdc42 input fixed and polarized throughout the simulation, the polarisome can be seen localizing away from the tip (see S5 Fig for details). We were able to stabilize Spa2 polarization in the tip by increasing the rate of recruitment of Bni1 by active Cdc42 in this fixed Cdc42 distribution setting (see S7 Fig for details). Taken together, these results would seem to indicate, at least for this particular model, that actin dynamics are largely insufficient to overcome the effect of the tip-shaped geometry. In this case, there is again a critical interaction between cell shape and the dynamics of a model of polarization, this time with the addition of actin dynamics.

## Discussion

In this paper we have shown that there can be a noticeable effect of geometry on the dynamics of certain reaction-diffusion models of polarization in yeast. Specifically, there seems to be a clear bias away from the tip of projection shaped geometries for a number of models tested. This is important when considered in the context of mating projection growth in yeast. When growing a mating projection, the cell establishes a spatial localization of proteins on the membrane, ultimately leading to actin cable formation and vesicle transport. The vesicles carry, among other proteins, cell wall modifying enzymes that change the material properties of the cell wall, leading to projection growth. During the growth of the projection *in vivo*, it is critical that the polarization cap stays at the tip of the projection to direct growth. However, our simulations suggest that current models of polarization could fail to capture this behavior due to a previously under-appreciated or unacknowledged effect of geometry on the dynamics of these polarization models. The main goal of this paper has been to quantify this effect for a variety of different polarization models and geometries.

We first demonstrated the effect complex geometries can have by simulating two different polarization models on realistic, tip-shaped geometries. For a mechanistic and detailed biochemical model of Cdc42 polarization, we showed that even when starting with polarized initial conditions in the tip of the projection, there is a tendency for the polarization cap to drift away from the tip (see Fig 3). This effect was further shown by running a simpler model of polarization on these same geometries and noticing again a bias away from the projection (see Fig 4). Typically, these models of polarization have been formulated as PDEs in simple geometries such as a line or sphere, thus the effect of complex geometry on the dynamics of the model has not appeared.

To understand this phenomenon further, we ran these models on a variety of tubular geometries in addition to the canonical projection geometries mentioned above. Interestingly, when simulated in a projection that was much longer than typically seen in WT yeast cells, the polarization cap would in fact stay close to the tip of the projection. This suggests there may be an interaction of length scales present in the problem. Namely, the polarization cap has a certain size in any given geometry. While currently it is not possible to a priori know the size of the polarization cap for an arbitrary geometry, the size of the polarization cap for a variety of shapes can be seen in Figs 1, 5 and 6. The size of the polarization cap will certainly be affected by the rates of reactions and diffusion in the model and the geometry itself. What is clear for the shorter projection geometries, seen in Fig 1, is that the polarization cap is of comparable size to the length of the projection. Whereas with longer projections, seen in Fig 5, the polarization cap is much smaller than the length of the projection. This is one possible explanation for the difference in stability between the two shapes. Specifically, it appears as though there are multiple metastable spatial locations for polarization in any geometry and the size of the polarization cap (which is related to the rates of reactions and diffusion) relative to these points may determine stability for a given initial site of polarization. Our simulations suggest that there is a complex interaction between the size of the polarization cap and the size of local features present in the geometry of the domain, for example the local radius of curvature or the length of the projection. Another related, yet distinct, phenomenon we considered here is the effect of changes in volume and density. We ran a model of Cdc42 polarization on a series of spheres of varying size, keeping either the number of molecules in the simulation or the density constant (see Fig 6). These results show that for large changes in volume and density, there can be significantly different dynamics for these models. The purpose of simulating polarization in spheres of varying radii was to understand the effects of changes in density independently from changes in overall shape. While a general theory for the stability of a given polarization model in an arbitrary domain is beyond the scope of this paper, the results we have presented already raise interesting biological questions.

While the main thrust of this paper is to elucidate the effects that complex geometries can have on mathematical models of polarization in yeast, we were curious to see whether similar effects could be observed experimentally. The main prediction we set out to test was that of the polarization of Cdc42 being spatially stable in a sphere, unstable in a short projection and stable in a long projection. To do this, we focused on a mutant cell line *cla4*Δ. These mutant cells can grow abnormally long projections, as Cla4 plays a vital role in septin formation. In an attempt to isolate the effect of geometry on Cdc42 polarization in the absence of actin cables and vesicle transport, the cells were grown in *α*-factor for varying times to achieve different lengths of projection growth and then LAT-A was added to disrupt actin cable formation, leaving the cells solely with Cdc42 reaction-diffusion dynamics for polarization. Next, cells were categorized as either spherical, short projection or long projection and fluorescent Ste20 (a reporter for active Cdc42) was monitored in time. As predicted by our simulations, the polarization cap was seen to be much more spatially stable in the spherical and long projection cells than in the shorter projection cells. While overall this is a qualitative result, it does suggest that current models of polarization could be lacking elements when more complex geometries are considered.

Based on our simulation and experimental results, one of the first questions that comes to mind is: how does the cell overcome this effect *in vivo*? The first natural thought might be that it is the actin cable network and vesicle transport that is providing the necessary reinforcement. As mentioned above, this is actually quite a difficult hypothesis to test computationally simply due to the wide range of approaches in the literature for mathematically modeling this process. We merged one model of actin polarization with a model of Cdc42 polarization to test if this could provide the necessary feedback to overcome the effect of geometry (see S2 and S3 Figs). Our results suggest that, at least for these particular models, actin dynamics are unlikely to overcome this effect of geometry. Again, as there are a wide variety of methods for modeling vesicle transport, it is still very possible that *in vivo* actin is contributing to overcoming the effect of geometry. One key feature that is typically neglected in models of actin cable formation is that the cables are in fact 1D structures embedded in a 3D space. Because the actin cables have a physical extent, they can be constrained by the geometry of the cell, providing a possible mechanism to overcome geometry effects. While there are methods to model such systems rigorously (see [39]), it is not common.

This discussion raises a broader issue. Polarization is an essential part of many biological processes in a wide variety of cell types. Often, when modeling polarization, the effects that complex geometry can have on polarization dynamics are not taken into account. Our results suggest not only that there is a qualitative and important effect on certain models of polarization, but also that these interactions can lead to valuable insights into the relevant biology that might have been overlooked otherwise. Moving forward, it may be necessary to consider the effect of geometry on reaction-diffusion dynamics when models of a given polarization process are being built.

## Materials and methods

### The reaction-diffusion master equation and computational details

As mentioned above, the RDME [40] is a mesoscopic model for spatial stochastic chemical reactions. It provides the time evolution of the probability distribution for the state of the system. First, the physical domain is partitioned into *K* nonoverlapping subvolumes or voxels, similar to numerical methods for PDEs. Molecules are taken to be point particles and the state of the system is the discrete number of molecules of each species for each of the voxels in the mesh. The computational mesh can either be a structured Cartesian grid or an unstructured triangular or tetrahedral mesh. Here, we focus on the unstructured case which will be discussed further below. Modeling the reaction-diffusion dynamics as a Markov process yields the following forward Kolmogorov equation for the time evolution of *p*(**x**, *t*) = *p*(**x**, *t*|**x**_0_, *t*_0_) (the probability that the system can be found in state **x** at time *t*, conditioned on the initial condition **x**_0_ at time *t*_0_)

$$\frac{\partial p(x,t)}{\partial t}=Rp(x,t)+Dp(x,t)$$ (1)

$$\begin{array}{ccc} Rp(x,t)= & & \sum_{i=1}^{K}\sum_{r=1}^{M}a_{ir}\left(x-\nu_{ir}\right)p\left(x-\nu_{ir},t\right)\\ & & -a_{ir}\left(x\right)p\left(x,t\right) \end{array}$$ (2)

$$\begin{array}{ccc} Dp(x,t)= & & \sum_{s=1}^{N}\sum_{i=1}^{K}\sum_{j=1}^{K}d_{sij}\left(x-\mu_{sij}\right)p\left(x-\mu_{sij},t\right)\\ & & -d_{sij}\left(x\right)p\left(x,t\right) \end{array}$$ (3)

where **x**_*i*⋅_ denotes the *i*-th row and **x**_⋅*j*_ denotes the *j*-th column of the *K* × *S* state matrix **x**, where *S* is the number of chemical species. The functions *a*_*ir*_(**x**_*i*_) define the propensity functions of the *M* chemical reactions, and *ν*_*ir*_ are stoichiometry vectors associated with the reactions. The propensity functions are defined such that *a*_*ir*_(**x**)Δ*t* is the probability that reaction *r* occurs in a small time interval of length Δ*t*. The stoichiometry vector *ν*_*ir*_ defines the rules for how the state changes when reaction *r* is executed. *d*_*ijk*_(**x**_*i*_) are propensities for the diffusion jump events, and *μ*_*ijk*_ are stoichiometry vectors for diffusion events. *μ*_*ijk*_ has only two non-zero entries, corresponding to the removal of one molecule of species *X*_*k*_ in voxel *i* and the addition of a molecule in voxel *j*. The propensity functions for the diffusion jumps, *d*_*ijk*_, are selected to provide a consistent and local discretization of the diffusion equation, or equivalently the Fokker-Planck equation for Brownian motion.

In most cases, the RDME is too high-dimensional to solve directly. Thus, algorithms have been developed that generate exact realizations of the Markov process described by the RDME, in a Monte Carlo fashion. One particularly efficient algorithm that we focus on for our implementation is the Next Subvolume Method (NSM) [21]. In this algorithm, the time to the next event in each voxel (either a chemical reaction or diffusion event) calculated by the Direct Method formulation of the SSA [17]. To identify in which voxel the event occurs, the algorithm uses the Next Reaction Method formulation of the SSA [41]. If it was a chemical reaction event that occurred, then only the voxel in which the event occurred needs to be updated, while if a diffusion event occured both the voxel where the molecule originated and the voxel where the molecule ended up need to be updated. The key to the efficiency of the NSM is the use of an event priority queue which gives a scaling of $O({\text{log}}_{2}\left(K\right))$ where *K* is the number of voxels in the mesh.

The use of unstructured meshes allows for complicated geometries in 3D to be more easily accommodated, such as the curved surfaces of cell membranes. PyURDME [23] (based on URDME [42]) is a software framework for simulation of the RDME on unstructured meshes that we have previously extended to time-dependent domains [24]. For the theoretical details of how to obtain mesoscopic diffusion constants on unstructured meshes, see [43]. Using the finite element package DOLFIN [44] we obtain the diffusion matrix for the system, from which we get the jump coefficients for individual voxels. The flexibility of simulating on unstructured meshes allows our method to handle complex domains in 3D, which is critical for our investigation into the effect of geometry on the dynamics of polarization models.

All models and simulation results presented in this work were built and performed using PyURDME and MOLNs [23]. Computational meshes for each geometry consisted of a discretization of both the cytoplasm and the membrane (the surface of the shape), allowing for diffusion both in the cytoplasm and on the membrane as required by the models used in this study. All reactions take place in voxels on the membrane for each geometry. These meshes were generated using Gmsh [45]. Sets of stochastic realizations for each model were run in parallel on a cluster of 64 machines. For simulations with polarized initial conditions (such as those in Fig 3), 20 realizations were run for a variety of different initial conditions. This is due to the fact that the specific configuration of the initial conditions could bias the dynamics of the model. To eliminate this bias and explore the full behavior of the model, initial conditions were rotated about the axis of symmetry (resulting in the apparent 4-fold symmetry in Fig 3 for example). What is important is that for any given polarized initial condition, there was a clear bias away from the tip of the projection, as discussed in the main text. One major difference in the simulation details and data analysis mentioned above is between the more detailed, mechanistic models of polarization (the S1 Model and the S3 Model) and the simplified Altschuler model (the S2 Model). This is due to a fundamental difference in dynamics between the two classes of models. While the mechanistic models reliably create one stable polarization cap (in the standard spherical geometry they were built for) the Altschuler model is more dynamic in time with clusters forming and breaking up. This is the main reason for the difference in simulation time for the two classes of models. All of the mechanistic model simulation results (found in Figs 3, 5 and 6 and all figures in the Supporting Information) were run for the same amount of time (1000s) as this is significantly longer than these models take to come to steady state while the Altschuler model simulation results (found in Fig 4) were run for much longer (100,000s) as, again, it is more dynamic in time and does not come to a steady state with one stable, fixed polarization cap. As far as the number of realizations is concerned, we ran enough realizations to clearly show the trend for each model. For example, the results in Fig 3 were run for 800 realizations to clearly show the manifold where the polarization caps were ending up while the results in Fig 5 were run for 400 realizations as the manifold was much easier see with fewer realizations.

Lastly, it is important to note we have no reason to believe that the effect of geometry on these models of polarization is an inherently stochastic phenomenon. That said, it is critical to note that two of the models used here (namely the simplified model of Cdc42 polarization, the S2 Model, and the model of actin polarization, the S3 Model) have been previously shown to rely on stochastic effects [5, 35]. Specifically, the simplified model from [35] was shown to only exhibit clustering (or polarization) when modeled stochastically as opposed to deterministically (at least for the parameters studied). Also, the model of actin polarization used, adapted from [5] was shown to replicate the experimental data of actin polarization better than the deterministic analog of the model through an effect that was dubbed “spatial stochastic amplification” in [5]. Thus, for these two models specifically it was necessary to model them stochastically for both the spherical and projection shaped geometries to faithfully replicate the dynamics of the model. As mentioned previously, though we don’t have any reason to believe that this effect of geometry on the dynamics of these models is itself a stochastic effect, we are showing an effect of geometry on models that rely on stochasticity. The other model used is the paper (the mechanistic model of Cdc42 polarization, the S1 Model, adapted from [4]) was the one model used that was originally formulated as a deterministic model. In theory, using a stochastic method to model the same set of reactions as a deterministic model will not be “missing” any important dynamics. Namely, the stochastic model will either replicate directly the deterministic results or there will be differences due to some stochastic effect (in which case the stochastic model will be more accurate if formulated correctly). For this particular case, our stochastic results for the spherical geometry do match the deterministic results from [4] well (although our models are not exactly equivalent as mentioned previously). One major concern with using stochastic methods is computational efficiency. Namely, the need to run several realizations can often increase the computational requirements of the simulation. We are uniquely positioned to deal with these issues as we have developed software to efficiently simulate stochastic models and leverage cloud computing infrastructure in the process [23]. With all of this in consideration, we found it more straightforward to simulate all of our models using stochastic methods for consistency. It would be interesting to replicate our experiments in a deterministic setting to see if there are any differences in behavior but it is nontrivial to solve these reaction-diffusion models as three-dimensional partial differential equations on irregular domains and we do not currently have a framework for doing this efficiently. In summary, while we have no reason to believe at this moment that the effect of geometry is a stochastic effect in itself, two of the models we have considered rely directly on stochastic effects and in theory modeling any of these reaction-diffusion systems stochastically will not miss any important dynamics. It will be interesting going forward to replicate our simulations in a deterministic setting and compare the results.

### Experimental setup

All yeast strains were derivatives of W303-1A and contained the *bar1*Δ mutation that prevents *α*-factor degradation by deletion of the Bar1 protease. Genetic techniques were performed per standard methods. All strains were cultured in YPD (yeast extract-peptone-dextrose) media supplemented with adenine. GFP-tagging was constructed by genomic integration using vectors amplified and targeted by PCR primers.

To experimentally test the computational predictions, we treated the *STE20-GFP cla4*Δ yeast strain CGY-021 with LAT-A (Invitrogen). LAT-A at a concentration of 50 *μ*M was added to cells exposed to *α*-factor (1 *μ*M) for 30 minutes (for spherical cells) or 90 minutes (for tip projected cells). These cells were imaged on slides for 30 minutes every 2 minutes after the addition of LAT-A. Images were acquired with a Nikon TE-300 inverted microscope using a 60x objective (NA = 1.4). Image analysis was manually performed using ImageJ.

The genotype of the strain CGY-021 is *MATa, can1-100, ade2-1, leu2-3,-112, his3-11,-15, trp1-1, ura3-1, bar1::hisG, ste20*Δ*::STE20-GFP-HIS5, cla4*Δ*::KAN^R^*

## Supporting information

S1 Fig Spherical coordinates of the center of active Cdc42 polarization for multiple realizations with random initial conditions. Here, the center of the polarization cap is tracked for four different shapes. Plotted is the theta and phi coordinates (explained in Fig 2) of the center of the polarization cap after 1000 seconds of simulation starting from randomly scattered initial conditions. Each point represents the result of one stochastic realization. The points of polarization for the sphere are completely random, as expected from these polarization models. In contrast, the cap is forming in a similar pattern to where the caps in Fig 1B drifted to for the three irregular shapes, hinting at a globally stable position for the polarization cap in these geometries. Interestingly, the last shape of a longer projection actually seems to be in between randomly polarized in the cell body and preferentially polarized at the neck of the projection. The dashed red lines here are a region of ±10° from the tip with the red square in the center representing the tip of the projection.
(TIF)

S2 Fig Spherical coordinates of the center of active Cdc42 polarization for multiple realizations with polarized initial conditions, for the combined Cdc42 and polarisome model. To initially test the hypothesis that the actin network and vesicle traffic could overcome the negative effect of the tip shaped geometry, we simulated a combined model of Cdc42 and actin polarization. As with previous simulations, starting from a polarized initial condition in the tip of the projection, the Cdc42 cap is seen to drift away from the tip. This is even with the added positive feedback from the polarisome to Cdc42. It should also be noted that the length scale of actin and Spa2 polarization is smaller than for Cdc42. While this isn’t definitive proof that actin isn’t helping to keep the polarization cap in the tip of the projection, it does show that for these reaction-diffusion models of Cdc42 and actin polarization, there is a persistent bias away from the tip.
(TIF)

S3 Fig Spherical coordinates of the center of Spa2 polarization for multiple realizations with polarized initial conditions, for the combined Cdc42 and polarisome model. These are the corresponding centers of Spa2 polarization for the results shown in S2 Fig.
(TIF)

S4 Fig Spherical coordinates of the center of active Cdc42 polarization for multiple realizations with random initial conditions, with constant density rather than constant molecule count. Here, we tested our results presented in S1 Fig by adjusting the molecule count to keep a constant density for each geometry (opposed to a constant molecule count). For these relatively small changes in total volume, the overall behavior of a bias away from the tip is preserved for both constant molecule and constant density.
(TIF)

S5 Fig Spherical coordinates of the center of Spa2 polarization for multiple realizations with polarized initial conditions, for the polarisome model with a fixed active Cdc42 distribution as input. These results are to be compared to the results presented in S2 and S3 Figs. Here the active Cdc42 profile is fixed and polarized in the tip of the geometry, rather than fully dynamic as above. This, presumably, would make it more likely for Spa2 to polarize in the tip as geometry is no longer having an effect on the Cdc42 dynamics yet the geometry still appears to have an effect on the polarisome. This further supports our general result of geometry having a significant impact on the dynamics of polarization.
(TIF)

S6 Fig Spherical coordinates of the center of active Cdc42 polarization for multiple realizations with polarized initial conditions and one visualization of drifting with diffusion in the cytoplasm *D*_*c*_ = 10 *μm*^2^ *s*^−1^. Here, we investigate the role of cytoplasmic diffusion on polarization in different geometries. Specifically, in addition to the cytoplasmic diffusion coefficient of *D*_*c*_ = 50 *μm*^2^ *s*^−1^ we have tested a variety of other diffusion coefficients and in particular here show results for *D*_*c*_ = 10 *μm*^2^ *s*^−1^ [46]. A: Spherical coordinates of the center of active Cdc42 polarization. While there is a difference in the stability for the slightly deformed geometry, the bias away from the tip for projection shaped geometries is still clear. B: Visualization of one realization of the active Cdc42 polarization cap over time in the longer projection geometry.
(TIF)

S7 Fig Spherical coordinates of the center of Spa2 polarization for multiple realizations with scattered initial conditions and a fixed active Cdc42 distribution as input and one visualization of the stabilization of polarization in a tip shaped geometry. These results are to be compared to the results presented in S2, S3 and S5 Figs. Here the active Cdc42 profile is fixed and polarized in the tip of the geometry, rather than fully dynamic as above. To investigate the possibility of stabilizing Spa2 polarization, we have increased the parameter *B*_*on*_ (which is the recruitment of Bni1 by active Cdc42) by a factor of 100. We see that this is in fact enough to stabilize Spa2 polarization in the tip of projection shaped geometries. A: Spherical coordinates of the center of Spa2 polarization with the increased value of *B*_*on*_. B: Visualization of one realization of the Spa2 polarization cap over time in the longer projection geometry.
(TIF)

S8 Fig *θ* versus distance from the tip of the projection of the active Cdc42 polarization for multiple realizations with polarized initial conditions. These results are to be compared to the results presented in Fig 3 of the main text. A: Here we plot *θ* versus the distance from the tip rather than the spherical coordinates of the polarization cap as above. B: A histogram of the distance away from the tip for each shape with multiple realizations. As in Fig 3, the difference between the spherical and the projection geometries is clear.
(TIF)

S1 Model Mechanistic model of Cdc42 polarization. The reactions and parameters for one model of Cdc42 polarization used in the main text. This set of reactions is adapted from a model of polarization during budding presented in [4] to account for the mating pheromone present during mating (through the presence of a uniform Gbg field) and a small negative feedback from Cla4.
(PDF)

S2 Model Simplified model of Cdc42 polarization. The reactions and parameters for a simplified model of Cdc42 polarization presented in [3].
(PDF)

S3 Model Mechanistic model of polarisome formation. The reactions and parameters for the model of actin dynamics adapted from [5] that was combined with the S1 Model in the main text to investigate the possibility of actin dynamics overcoming the effect of geometry on polarization. The model originally presented in [5] was formulated on a 1D domain. We have adapted these parameters and fit them in a similar way as was presented in [5] for a sphere.
(PDF)

S1 Movie Time-lapse of of GFP-tagged Ste20 in a spherical cell. Here is a time-lapse of GFP tagged Ste20 (a reporter for active Cdc42) for a spherical cell after the addition of LAT-A. This cell corresponds with the results shown in Fig 7B. The polarization cap can be seen to be spatially stable in this case.
(AVI)

S2 Movie Time-lapse of of GFP-tagged Ste20 in a cell with a short projection. Here is a time-lapse of GFP tagged Ste20 (a reporter for active Cdc42) for a cell with a short projection after the addition of LAT-A. This cell corresponds with the results shown in Fig 7B. The polarization cap can be seen to be spatially unstable in this case.
(AVI)

S3 Movie Time-lapse of of GFP-tagged Ste20 in a cell with a long projection. Here is a time-lapse of GFP tagged Ste20 (a reporter for active Cdc42) for a cell with a long projection after the addition of LAT-A. This cell corresponds with the results shown in Fig 7B. The polarization cap can be seen to be spatially stable in this case.
(AVI)
