# Supplementary figures and images for: The effect of cell geometry on polarization in budding yeast
Source: PLoS Comput Biol. 2018 Jun 11;14(6):e1006241. doi: 10.1371/journal.pcbi.1006241 (PMC6013239; doi:10.1371/journal.pcbi.1006241)

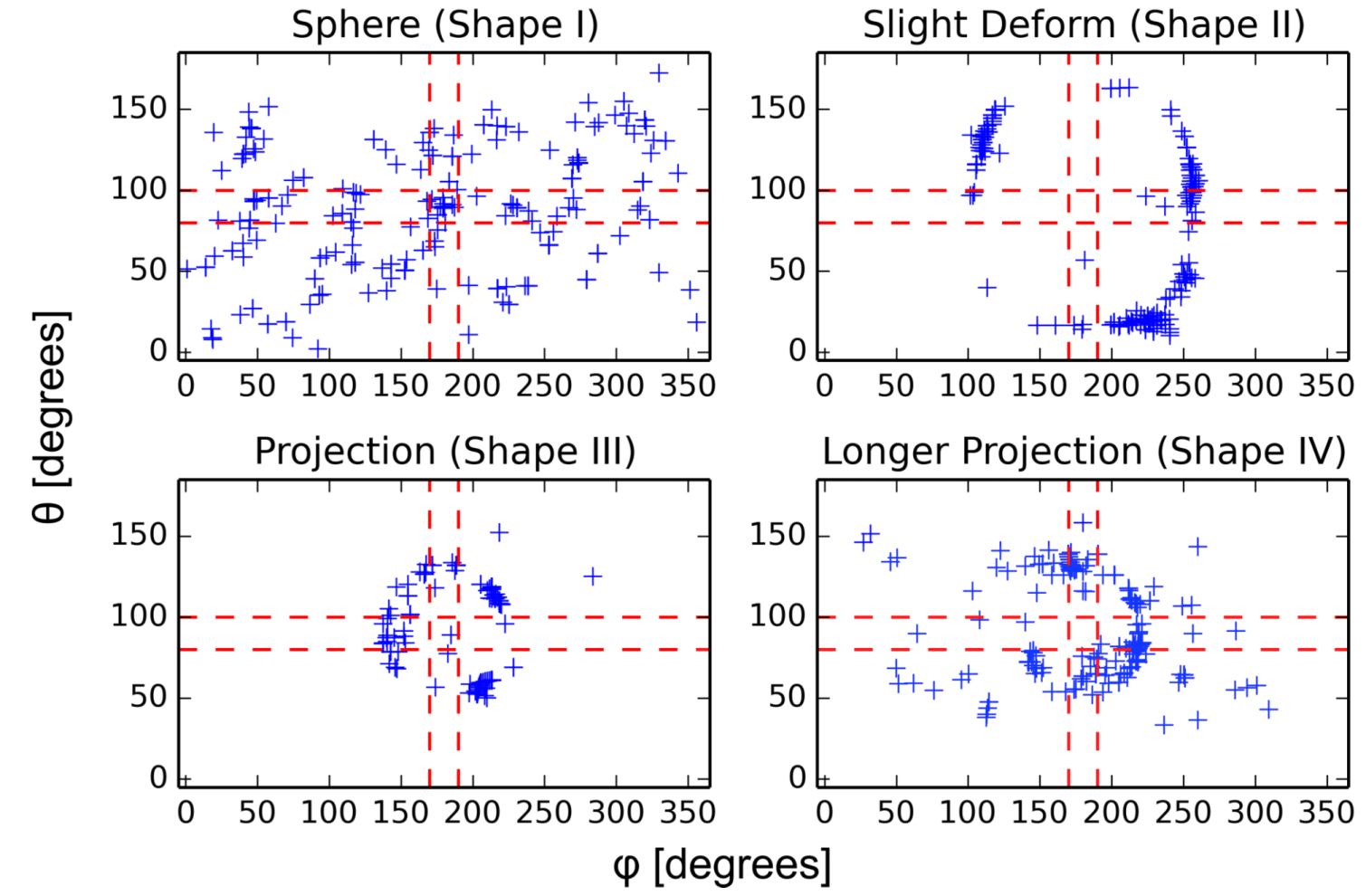

Supplement: S1 Fig — Here, the center of the polarization cap is tracked for four different shapes. Plotted is the theta and phi coordinates (explained in Fig 2) of the center of the polarization cap after 1000 seconds of simulation starting from randomly scattered initial conditions. Each point represents the result of one stochastic realization. The points of polarization for the sphere are completely random, as expected from these polarization models. In contrast, the cap is forming in a similar pattern to where the caps in Fig 1B drifted to for the three irregular shapes, hinting at a globally stable position for the polarization cap in these geometries. Interestingly, the last shape of a longer projection actually seems to be in between randomly polarized in the cell body and preferentially polarized at the neck of the projection. The dashed red lines here are a region of ±10° from the tip with the red square in the center representing the tip of the projection. (TIF) [file pcbi.1006241.s001.tif]

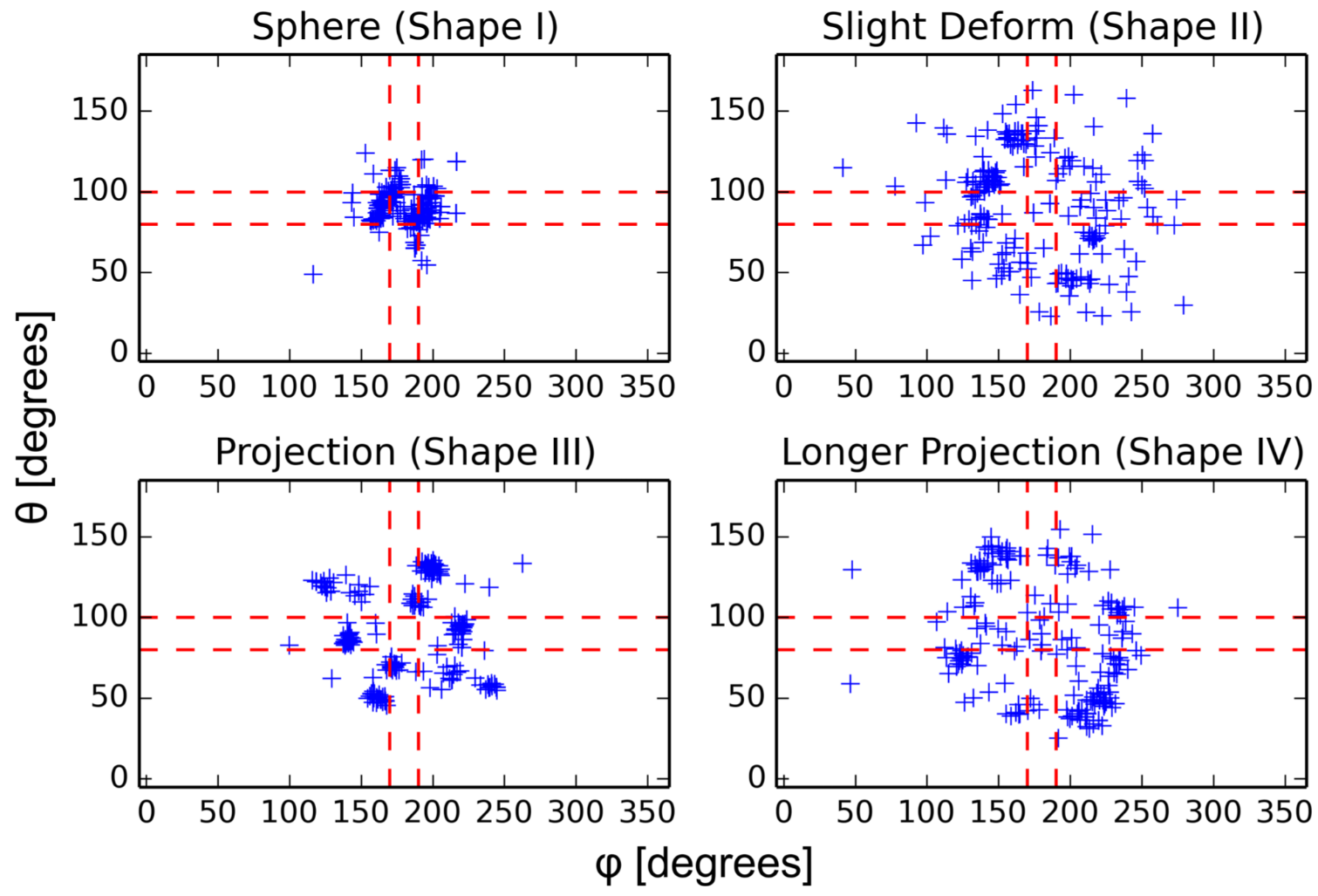

Supplement: S2 Fig — To initially test the hypothesis that the actin network and vesicle traffic could overcome the negative effect of the tip shaped geometry, we simulated a combined model of Cdc42 and actin polarization. As with previous simulations, starting from a polarized initial condition in the tip of the projection, the Cdc42 cap is seen to drift away from the tip. This is even with the added positive feedback from the polarisome to Cdc42. It should also be noted that the length scale of actin and Spa2 polarization is smaller than for Cdc42. While this isn’t definitive proof that actin isn’t helping to keep the polarization cap in the tip of the projection, it does show that for these reaction-diffusion models of Cdc42 and actin polarization, there is a persistent bias away from the tip. (TIF) [file pcbi.1006241.s002.tif]

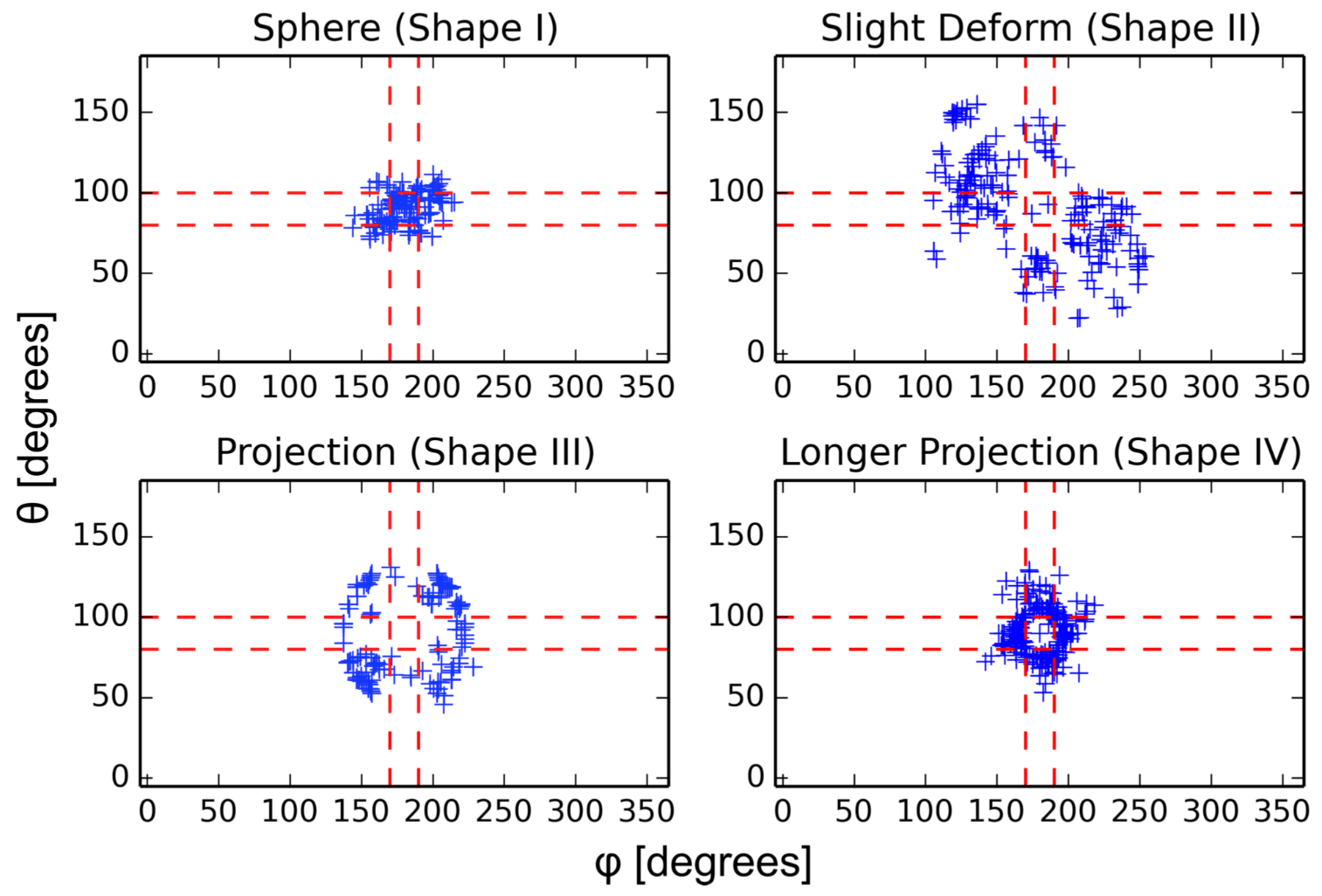

Supplement: S3 Fig — These are the corresponding centers of Spa2 polarization for the results shown in S2 Fig. (TIF) [file pcbi.1006241.s003.tif]

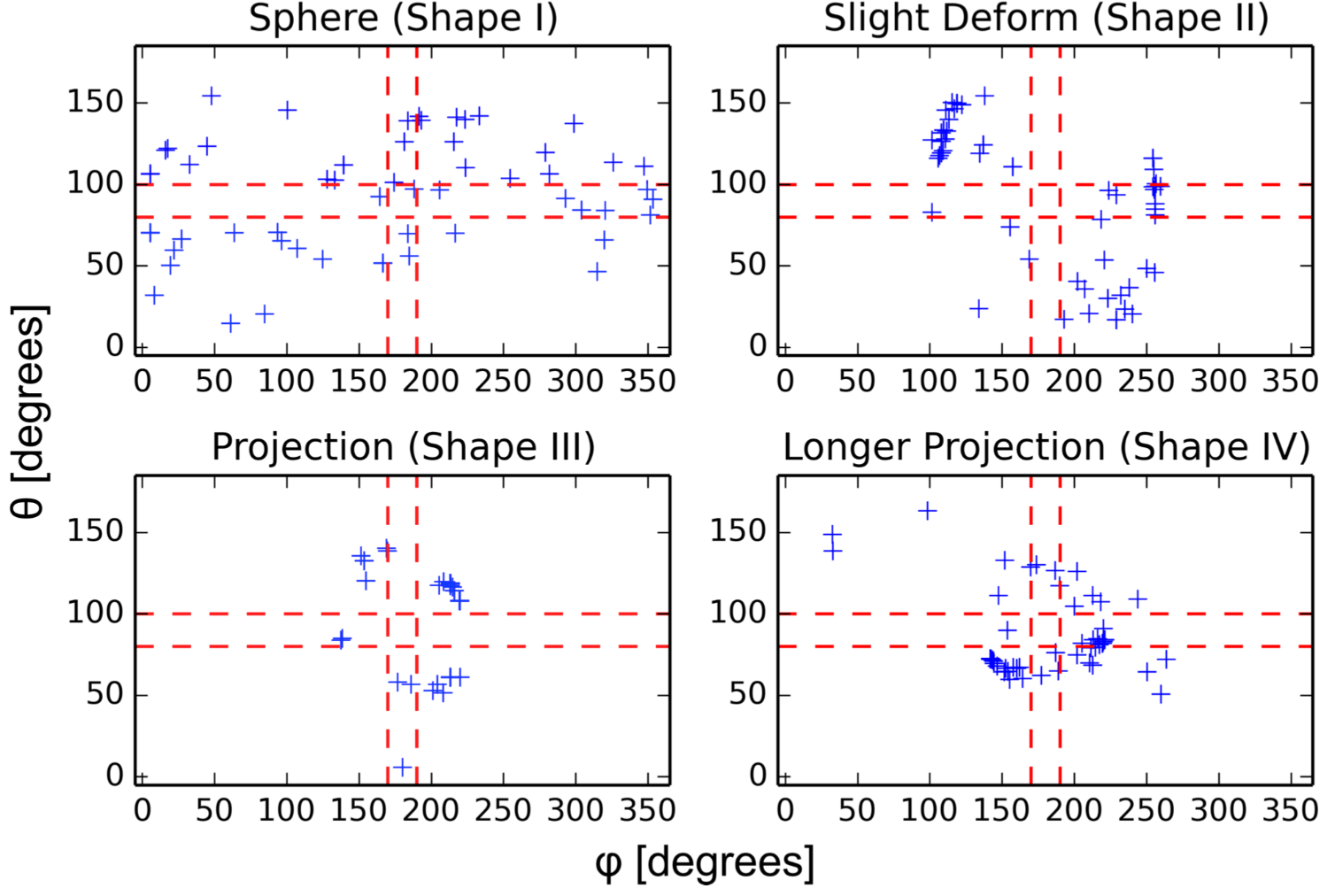

Supplement: S4 Fig — Here, we tested our results presented in S1 Fig by adjusting the molecule count to keep a constant density for each geometry (opposed to a constant molecule count). For these relatively small changes in total volume, the overall behavior of a bias away from the tip is preserved for both constant molecule and constant density. (TIF) [file pcbi.1006241.s004.tif]

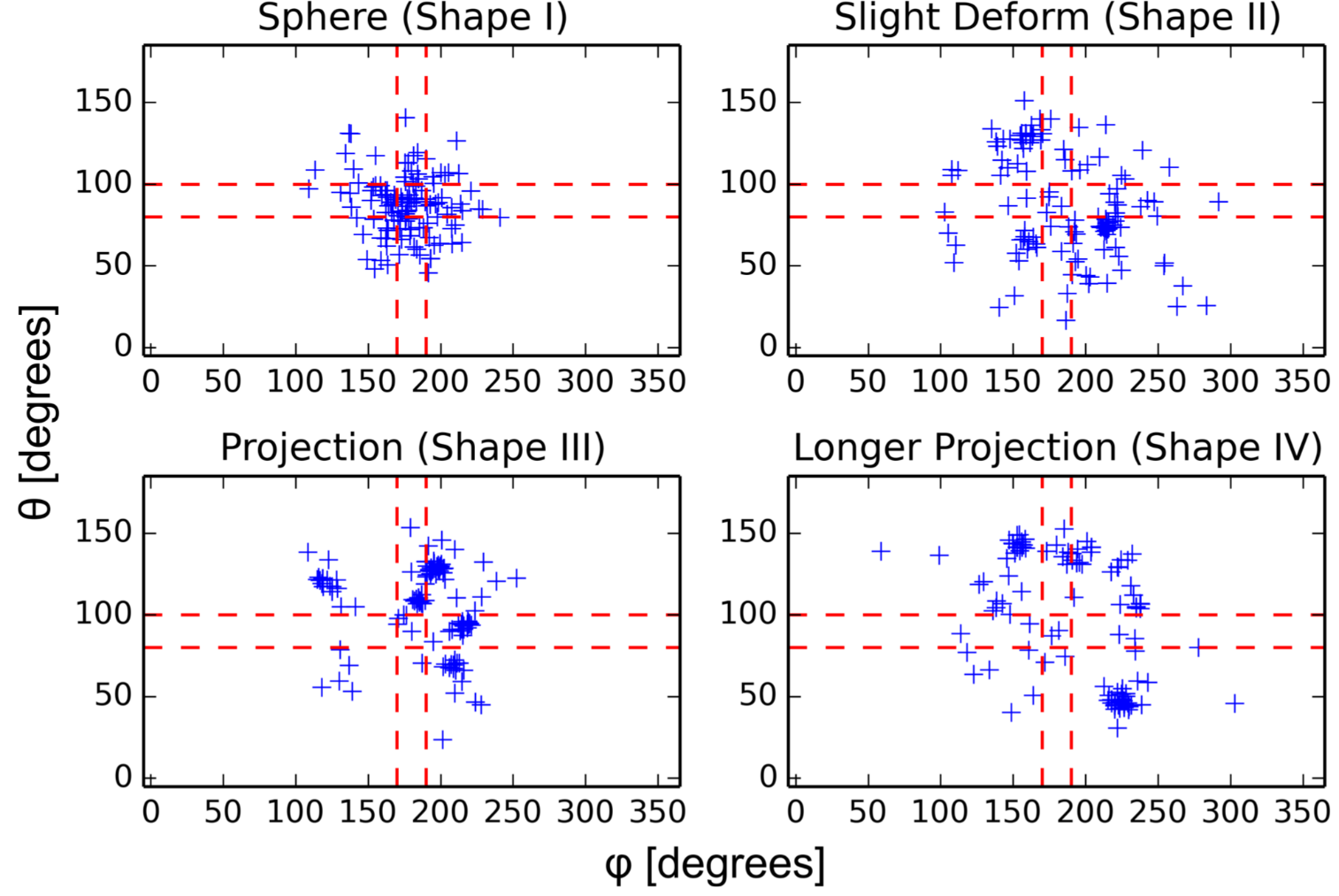

Supplement: S5 Fig — These results are to be compared to the results presented in S2 and S3 Figs. Here the active Cdc42 profile is fixed and polarized in the tip of the geometry, rather than fully dynamic as above. This, presumably, would make it more likely for Spa2 to polarize in the tip as geometry is no longer having an effect on the Cdc42 dynamics yet the geometry still appears to have an effect on the polarisome. This further supports our general result of geometry having a significant impact on the dynamics of polarization. (TIF) [file pcbi.1006241.s005.tif]

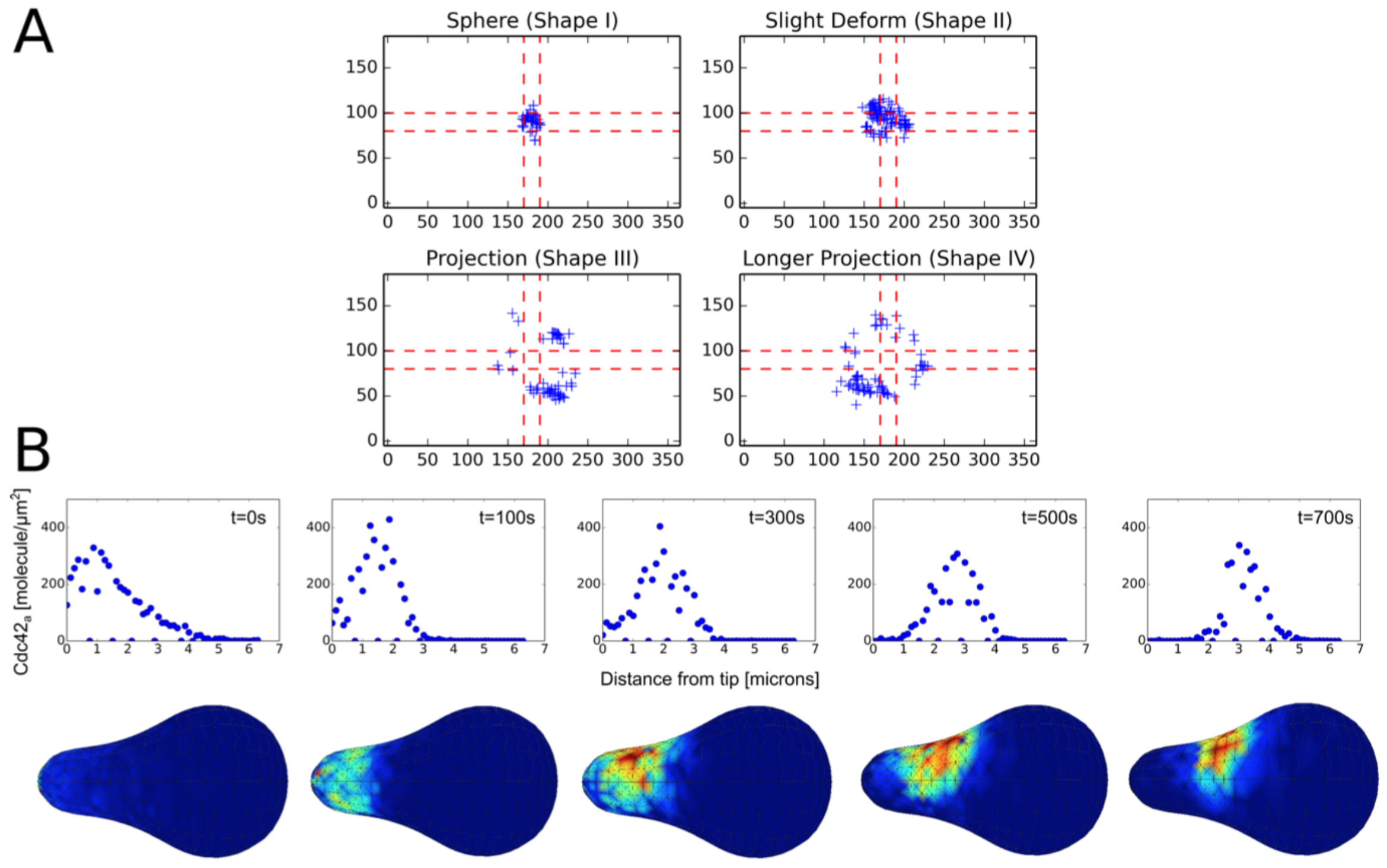

Supplement: S6 Fig — Here, we investigate the role of cytoplasmic diffusion on polarization in different geometries. Specifically, in addition to the cytoplasmic diffusion coefficient of Dc = 50 μm2 s−1 we have tested a variety of other diffusion coefficients and in particular here show results for Dc = 10 μm2 s−1 [46]. A: Spherical coordinates of the center of active Cdc42 polarization. While there is a difference in the stability for the slightly deformed geometry, the bias away from the tip for projection shaped geometries is still clear. B: Visualization of one realization of the active Cdc42 polarization cap over time in the longer projection geometry. (TIF) [file pcbi.1006241.s006.tif]

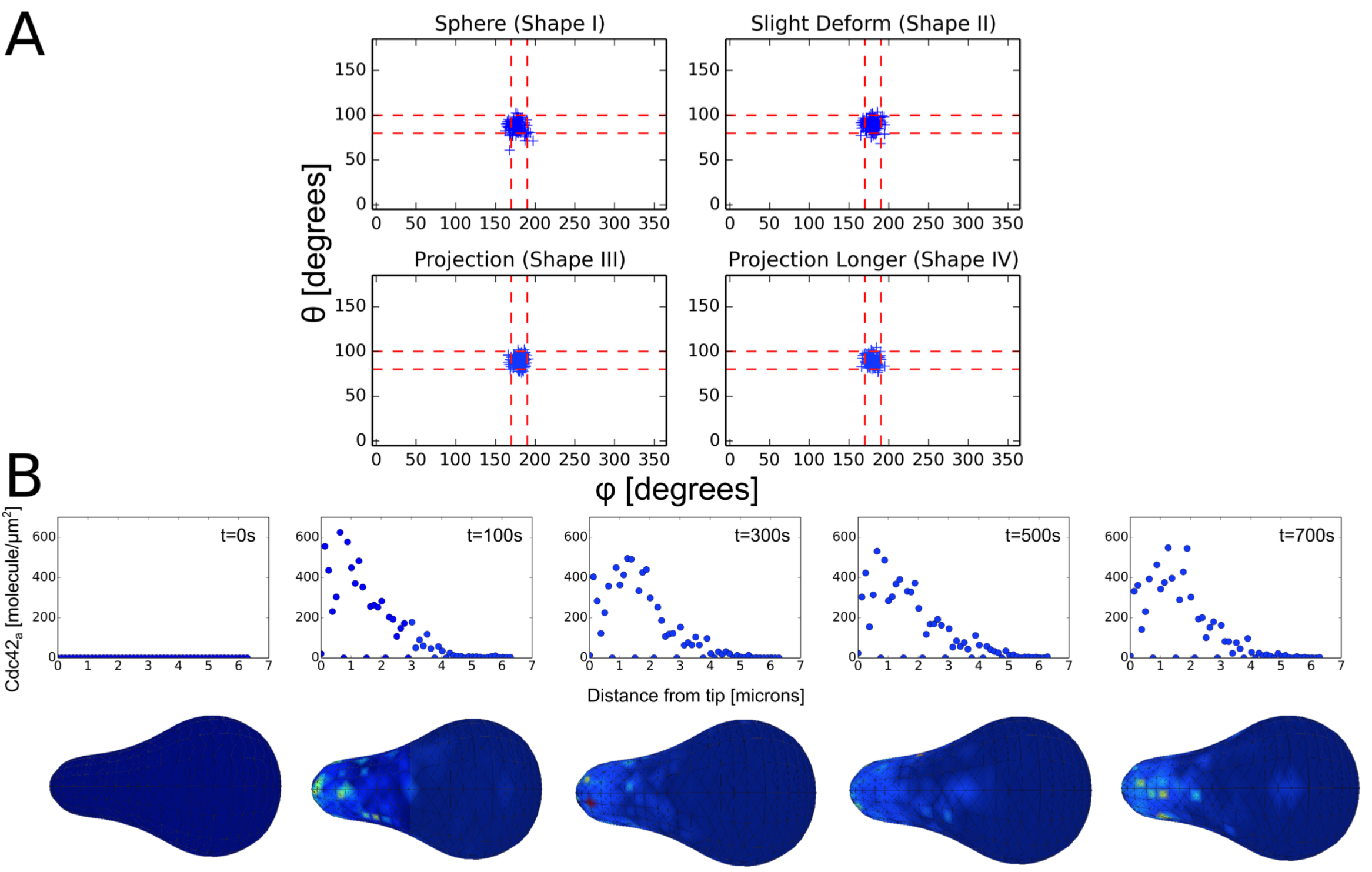

Supplement: S7 Fig — These results are to be compared to the results presented in S2, S3 and S5 Figs. Here the active Cdc42 profile is fixed and polarized in the tip of the geometry, rather than fully dynamic as above. To investigate the possibility of stabilizing Spa2 polarization, we have increased the parameter Bon (which is the recruitment of Bni1 by active Cdc42) by a factor of 100. We see that this is in fact enough to stabilize Spa2 polarization in the tip of projection shaped geometries. A: Spherical coordinates of the center of Spa2 polarization with the increased value of Bon. B: Visualization of one realization of the Spa2 polarization cap over time in the longer projection geometry. (TIF) [file pcbi.1006241.s007.tif]

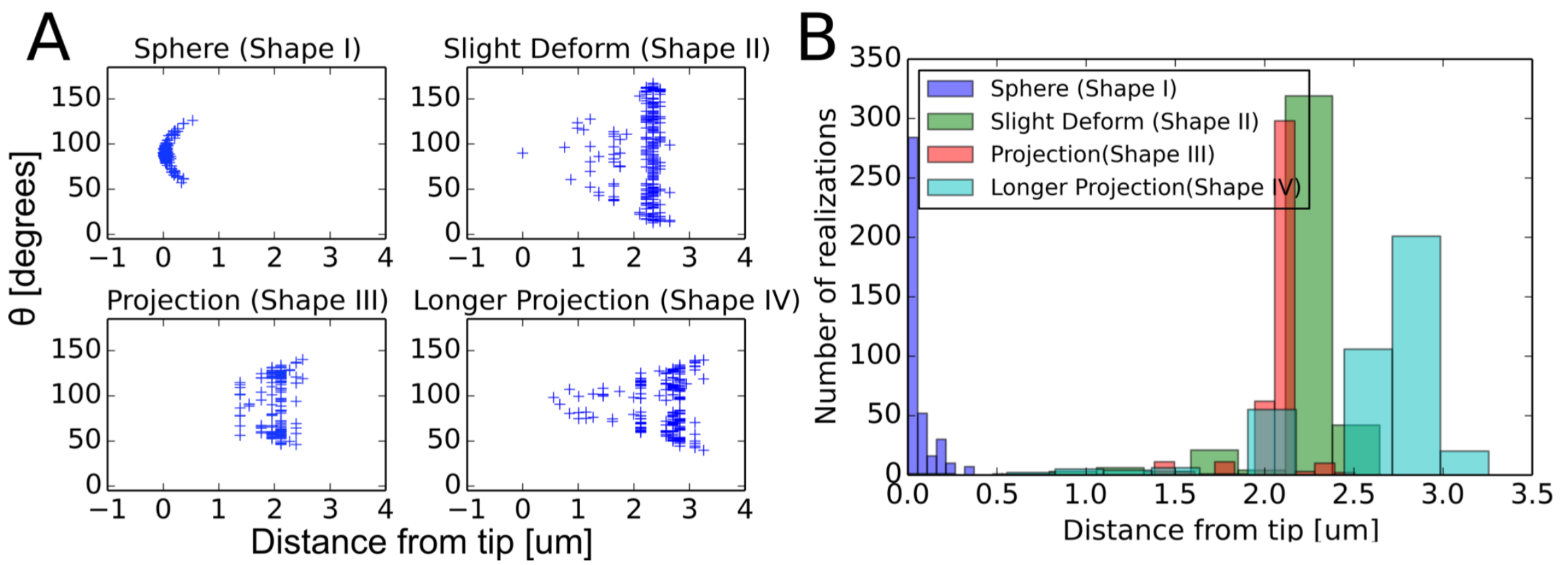

Supplement: S8 Fig — These results are to be compared to the results presented in Fig 3 of the main text. A: Here we plot θ versus the distance from the tip rather than the spherical coordinates of the polarization cap as above. B: A histogram of the distance away from the tip for each shape with multiple realizations. As in Fig 3, the difference between the spherical and the projection geometries is clear. (TIF) [file pcbi.1006241.s008.tif]
