# Supplementary material for: The effect of cell geometry on polarization in budding yeast
Source: PLoS Comput Biol. 2018 Jun 11;14(6):e1006241. doi: 10.1371/journal.pcbi.1006241 (PMC6013239; doi:10.1371/journal.pcbi.1006241)
Supplement: S1 Model — The reactions and parameters for one model of Cdc42 polarization used in the main text. This set of reactions is adapted from a model of polarization during budding presented in [4] to account for the mating pheromone present during mating (through the presence of a uniform Gbg field) and a small negative feedback from Cla4. (PDF) [file pcbi.1006241.s009.pdf]

S1 Model Mechanistic model of Cdc42 polarization.

$$Cdc24_c + Gbg \xrightarrow{\delta_1} Cdc24_m + Gbg$$
$$Bem1_m + Cdc24_c \xrightarrow{\delta_1} Bem1_m + Cdc24_m$$
$$Cdc24_m \xrightarrow{\delta_2} Cdc24_c$$
$$Cdc24_m + Cdc42GDP_m \xrightarrow{\alpha_1} Cdc24_m + Cdc42GTP_m$$
$$Cdc42GTP_m \xrightarrow{\alpha_3} Cdc42GDP_m$$
$$Bem1_c + Cdc42GTP_m \xrightarrow{\gamma_1} Bem1_m + Cdc42GTP_m$$
$$Bem1_m \xrightarrow{\gamma_2} Bem1_c$$
$$Cdc42GDP_c \xrightarrow{\beta_2} Cdc42GDP_m$$
$$Cdc42GDP_m \xrightarrow{\beta_3} Cdc42GDP_c$$
$$Cdc24_m + Cdc42GDP_c \xrightarrow{\beta_1} Cdc24_m + Cdc42GTP_m$$
$$Cdc24_m + Cla4_a \xrightarrow{k_{24d}} Cdc24_c + Cla4_a$$
$$Cla4_m + Cdc42GTP_m \xrightarrow{k_{Cla4a}} Cla4_a + Cdc42GTP_m$$
$$Cla4_a \xrightarrow{k_{Cla4d}} Cla4_m$$

Parameter	Value	Description	Source
D_m	0.0053 $\mu m^2 s^{-1}$	Diffusion constant on membrane	[5]
D_c	50 $\mu m^2 s^{-1}$	Diffusion constant in cytoplasm	See Fig S6
R	2 μm	Radius of cell	[5]
N_{42}	3000	Total number of Cdc42 molecules	[4]
N_B	3000	Total number of Bem1 molecules	[38]
N_{24}	1000	Total number of Cdc24 molecules	[4]
α_1	0.2 $\mu m^2 s^{-1}$	Activation of Cdc42 by Cdc24 (membrane)	[4]
α_3	1 s^{-1}	Deactivation of Cdc42	[4]
β_1	0.266 $\mu m^3 s^{-1}$	Activation of Cdc42 by Cdc24 (cytoplasm)	[4]
β_2	0.28 $\mu m s^{-1}$	Attachment of Cdc42 to membrane	[4]
β_3	1 s^{-1}	Detachment of Cdc42 from membrane	[4]
γ_1	0.2667 $\mu m^3 s^{-1}$	Bem1 recruitment by Cdc42	[4]
γ_2	0.35 s^{-1}	Detachment of Bem1 from membrane	[4]
δ_1	0.00297 $\mu m^3 s^{-1}$	Recruitment of Cdc24 by Gbg	[4]
δ_2	0.35 s^{-1}	Detachment of Cdc24 from membrane	[4]
k_{24d}	0.000033 $\mu m^3 s^{-1}$	Detachment of Cdc24 via Cla4	[38]
k_{Cla4a}	0.006 s^{-1}	Activation of Cla4 by Cdc42	[38]
k_{Cla4d}	0.01 s^{-1}	Deactivation of Cla4	[38]
