# Supplementary material for: The effect of cell geometry on polarization in budding yeast
Source: PLoS Comput Biol. 2018 Jun 11;14(6):e1006241. doi: 10.1371/journal.pcbi.1006241 (PMC6013239; doi:10.1371/journal.pcbi.1006241)
Supplement: S2 Model — The reactions and parameters for a simplified model of Cdc42 polarization presented in [3]. (PDF) [file pcbi.1006241.s010.pdf]

S2 Model. Simplified model of Cdc42 polarization.

$$Cdc42_c \xrightarrow{k_{on}} Cdc42_m$$

$$Cdc42_m \xrightarrow{k_{off}} Cdc42_c$$

$$Cdc42_c + Cdc42_m \xrightarrow{k_{fb}} 2Cdc42_m$$

Parameter	Value	Description	Source
k_{on}	$0.0001/60\ s^{-1}$	Spontaneous on rate	[3]
k_{off}	$9.0/60\ s^{-1}$	Spontaneous off rate	[3]
k_{fb}	$10.0/60\ \mu m^3 s^{-1}$	Feedback rate	[3]
