# Supplementary material for: The effect of cell geometry on polarization in budding yeast
Source: PLoS Comput Biol. 2018 Jun 11;14(6):e1006241. doi: 10.1371/journal.pcbi.1006241 (PMC6013239; doi:10.1371/journal.pcbi.1006241)
Supplement: S3 Model — The reactions and parameters for the model of actin dynamics adapted from [5] that was combined with the S1 Model in the main text to investigate the possibility of actin dynamics overcoming the effect of geometry on polarization. The model originally presented in [5] was formulated on a 1D domain. We have adapted these parameters and fit them in a similar way as was presented in [5] for a sphere. (PDF) [file pcbi.1006241.s011.pdf]

S3 Model   Mechanistic model of polarisome formation.

$$Bni1_c + Cdc42GTP_m \xrightarrow{B_{on}} Bni1_m + Cdc42GTP_m$$
$$Bni1_m \xrightarrow{B_{off}} Bni1_c$$
$$Actin_c + Bni1_m \xrightarrow{A_{on}} Actin_m + Bni1_m$$
$$Actin_m \xrightarrow{A_{off}} Actin_c$$
$$Spa2_c + Actin_m \xrightarrow{S_{on}} Spa2_m + Actin_m$$
$$Spa2_m \xrightarrow{S_{off}} Spa2_c$$
$$Bni1_c + Spa2_m \xrightarrow{B_{fb}} Bni1_m + Spa2_m$$

Added feedback between Cdc42 polarization and polarisome:

$$Cdc42GDP_c + Actin_m \xrightarrow{\beta_1} Cdc42GTP_m + Actin_m$$
$$Cdc42GDP_m + Actin_m \xrightarrow{\beta_1} Cdc42GTP_m + Actin_m$$

Parameter	Value	Description
D_m	0.0053 $\mu m^2 s^{-1}$	Diffusion constant on membrane
D_a	0.0 $\mu m^2 s^{-1}$	No actin diffusion on membrane
D_c	50 $\mu m^2 s^{-1}$	Diffusion constant in cytoplasm
R	2 μm	Radius of cell
N_B	1000	Total number of Bni1 molecules
N_S	5000	Total number of Spa2 molecules
N_A	40	Total number of Actin cables
B_{on}	0.000256 $\mu m^3 s^{-1}$	Recruitment of Bni1 by Cdc42
B_{off}	22.5 s^{-1}	Detachment of Bni1 from membrane
S_{on}	4.55 $\mu m^3 s^{-1}$	Recruitment of Spa2 by Actin
S_{off}	0.35 s^{-1}	Detachment of Spa2 from membrane
A_{on}	0.197 $\mu m^3 s^{-1}$	Recruitment of Actin by Bni1
A_{off}	$1.57 * 500/(500 + Spa2_m)$	Detachment of Actin from membrane
B_{fb}	0.0304 $\mu m^3 s^{-1}$	Recruitment of Bni1 by Spa2
β_1	0.266 $\mu m^3 s^{-1}$	Recruitment of Cdc42 by Actin
